# VarNMF: non-negative probabilistic factorization with source variation

**DOI:** 10.1093/bioinformatics/btae758

**Published:** 2024-12-28

**Authors:** Ela Fallik, Nir Friedman

**Affiliations:** School of Computer Science and Engineering, The Hebrew University of Jerusalem, Jerusalem, 9190401, Israel; Lautenberg Center for Immunology and Cancer Research, Faculty of Medicine, The Hebrew University of Jerusalem, Jerusalem, 9112102, Israel; School of Computer Science and Engineering, The Hebrew University of Jerusalem, Jerusalem, 9190401, Israel; Lautenberg Center for Immunology and Cancer Research, Faculty of Medicine, The Hebrew University of Jerusalem, Jerusalem, 9112102, Israel

## Abstract

**Motivation:**

Non-negative matrix factorization (NMF) is a powerful tool often applied to genomic data to identify non-negative latent components that constitute linearly mixed samples. It is useful when the observed signal combines contributions from multiple sources, such as cell types in bulk measurements of heterogeneous tissue. NMF accounts for two types of variation between samples — disparities in the proportions of sources and observation noise. However, in many settings, there is also a non-trivial variation between samples in the contribution of each source to the mixed data. This variation cannot be accurately modeled using the NMF framework.

**Results:**

We present VarNMF, a probabilistic extension of NMF that explicitly models this variation in source values. We show that by modeling sources as non-negative distributions, we can recover source variation directly from mixed samples without observing any of the sources directly. We apply VarNMF to a cell-free ChIP-seq dataset of two cancer cohorts and a healthy cohort, demonstrating that VarNMF provides a better estimation of the data distribution. Moreover, VarNMF extracts cancer-associated source distributions that decouple the tumor characteristics from the amount of tumor contribution, and identify patient-specific disease behaviors. This decomposition highlights the inter-tumor variability that is obscured in the mixed samples.

**Availability and implementation:**

Code is available at https://github.com/Nir-Friedman-Lab/VarNMF.

## 1 Introduction

The last few decades have brought great advances in DNA sequencing technologies, facilitating the collection of rich and diverse genomic datasets (Marguerat and Bähler 2010, Kimura 2013). In many applications, the sequenced sample represents an aggregate of multiple sources. For example, a liver tissue sample contains hepatocytes, but also endothelial cells and multiple types of immune cells. Similarly, a cancer biopsy contains tumor cells, but also a variety of other cell types from the surrounding tissue (Haider *et al.* 2020). Therefore, the signals obtained from such samples represent mixed information from the multitude of cell types that are present in the physical sample. In many settings, separating the sources’ contribution to the mixed signal helps us gain insights into the underlying processes. For example, biopsies from two cancer patients may differ in the relative proportion of tumor cells, but also in the tumor-contributing signal itself: certain genes can exhibit variation in signal within the tumor cells of the two patients (Rudin *et al.* 2019). Ideally, we want to distinguish between these two types of deviations.

Our motivation for examining the separation of mixed signals stems from the analysis of genomic data from an assay we recently introduced — *cell-free chromatin immunoprecipitation followed by sequencing* (cfChIP-seq, Supplementary Fig. S6, Sadeh *et al.* 2021). This assay is performed on plasma (the cell-free portion of blood) and captures DNA fragments marked with a specific epigenetic modification called H3K4me3, which is associated with active and poised promoters (Heintzman *et al.* 2007). These cell-free DNA fragments in the plasma originate from dying cells of various sources — cell types or tissues across the body — according to the cell type death proportions in the individual (Aucamp *et al.* 2018). The chosen modification marks DNA fragments that were located in active genes in the original cells (Soares *et al.* 2017). Therefore, the signal from each of these cell-types is tightly coordinated with gene activity. Decomposing the mixed signal into individual components would potentially identify gene-related activity in each sub-population of cells contributing to it (e.g. tumor cells, immune cells). This is crucial for interpreting complex samples.

The challenge of decomposing mixed signals has been approached from many directions (see review in Shen-Orr and Gaujoux 2013). Most previous works rely on reference data — a molecular characterization of potential contributing sources — used for estimating and quantifying the proportion of each source in the mixed sample. By relying only on previously characterized sources, these methods are unable to identify new or inaccessible sources of signal. Additionally, these characterizations are typically based on direct observations from isolated sources, which in many cases are infeasible to obtain. Moreover, the isolation process (e.g. physical cell separation and sorting) introduces technical biases, making the estimated characterizations non-transferable.

A different approach to decomposition is a data-driven approach of matrix factorization, employing algorithms such as principal component analysis (PCA) (Jolliffe and Cadima 2016) and independent component analysis (ICA) (Comon 1994). However, since the sequencing signal is based on counts of molecules, both the mixed signal and the sources contributing to it are non-negative. Therefore, a more natural model is the *non-negative matrix factorization* (NMF) model (Lee and Seung 2000), which decomposes the non-negative mixed signal V (an N×M matrix) into two low-rank non-negative matrices:

(1) V≈W·H

where $H_{k}\in R_{\geq 0}^{M}$ represents the kth source as a constant component, and each sample i=1,…,N is the result of linearly mixing these components with weights $W\left[i\right]\in R_{\geq 0}^{K}$ plus a “noise” term. NMF serves as a powerful tool for capturing underlying structure in mixed samples and identifying new sources of signal. Beyond sequencing data, it is also relevant to multiple other decomposition problems (e.g. Lee and Seung 1999, Smaragdis and Brown 2003, Arora *et al.* 2012).

**Figure 1.**
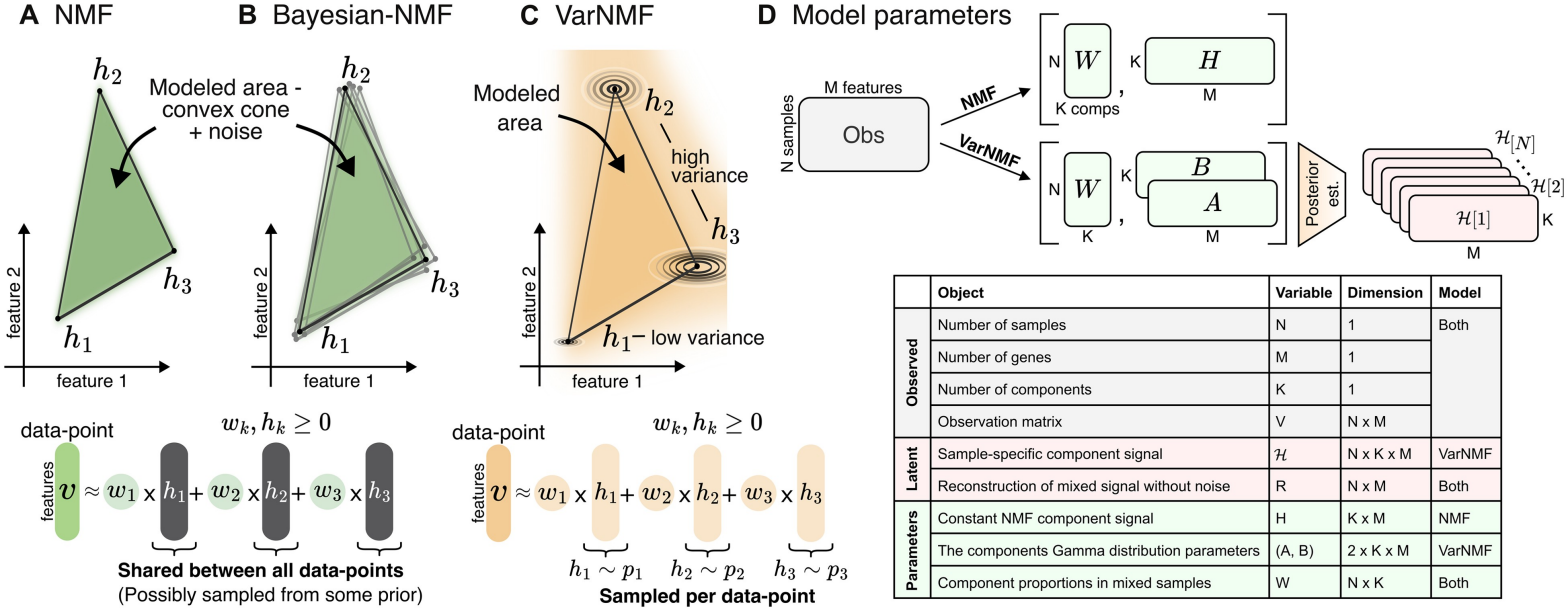
(A–C) Illustrative examples of mixed samples with K = 3 non-negative components (in black) and two representative features. (A) The NMF model assumptions allow us to model a convex cone between the constant components $h_{1},h_{2},h_{3}$, plus some noise. (B) In Bayesian-NMF there is a prior over the location of $h_{1},h_{2},h_{3}$, but the modeled area is still a convex cone. (C) In contrast, accounting for Type III variation in the sources means that each data point has its own instantiation of source contribution, sampled from the corresponding component distributions. This results in a wider modeled area. Separating the sources from mixed samples using NMF in this scenario will result in a cone that is wider than the original sources that created the data. (D) Details of the observed variables (grey), model parameters (green), and latent variables (red) – variables that are integrated into the model—for NMF and VarNMF, and the dimensions of each object.

In the NMF model, variation between samples can originate from observation noise — Type I variation — and from the mixing proportions W* —* Type II variation (Fig. 1A and B). Yet, in many applications, including cfChip-seq data, there are differences that are not explained entirely by these two factors: In Sadeh *et al.* (2021), cfChIP-seq was applied to a large number of subjects. The results display differences between samples in the signal originating from specific cell types, that are not explained by the proportions of cell-death (Type II variation) or observation noise (Type I). For example, in a cohort of patients with liver diseases, there are clear differences between the patients’ samples and the healthy baseline (Fig. 5 of Sadeh *et al.* 2021). Accounting for the increased cell-death proportions of liver tissue in the patients explains some differences, but not all, even when focusing on liver-specific genes. This suggests an additional *Type III variation* between samples in the liver signal itself. A more recent paper from our group (Fialkoff *et al.* 2023) also identified a specific variation in liver signal in an auto-immune liver disease. These results indicate that the liver signal does not fit a constant characterization. Rather, it presents variability in some genes’ signals between subjects and over time. Such differences in the state of cells cannot be captured or reasoned about in NMF and its existing extensions.

To account for this Type III variation — variation in sources signal between samples — we introduce a probabilistic graphical model we call VarNMF, where each source k is modeled as a non-negative distribution $p_{k}$ instead of a constant component vector (Fig. 1C), and the parameters of these distributions and the mixing weights are estimated from data using an *expectation maximization* (EM) procedure (Dempster *et al.* 1977). This modeling also allows us to estimate the *posterior profiles —* the signal contributed by every source to each particular sample — and to investigate unique behaviors that differ from the expected contribution.

Next, we formally present the NMF and VarNMF models, discuss algorithms to estimate parameters for both models, and compare their performances on synthetic data. Specifically, unlike some decomposition works, we argue that the focus should be on accurate estimation of the sources. This includes assessing the accuracy of the estimated distribution means, and evaluating how well VarNMF captures Type III variation (via the per-sample posterior profiles). Additionally, in biological settings, we are not only interested in fitting the given training data, but mostly in inferring biological insights that generalize to new samples. Therefore, we also examine how effectively the learned sources decompose new data points from the same distribution. Finally, we apply both models to real-world cfChIP-seq data to test how VarNMF confronts the problem presented above, and what is the extent of its interpretability.

## 2 Materials and methods

### 2.1 Background and related works

#### 2.1.1 Notations

We use i as sample index, and X[i] as the variable X in the ith sample. We use j as the feature (gene) index, and k as a component index. We denote V data matrix of N samples with M features. Each row is a sample $V\left[i\right]\in R_{\geq 0}^{M}$, which is a mix of signals from K sources according to the weights $W\left[i\right]\in R_{\geq 0}^{K}$. The sources are represented as K non-negative M-dimensional vectors $H_{k}$ or distributions $p_{k}$ (depending on the model, see below). In the latter case, we regard each sample-specific instance of the component distribution $p_{k}$ as a non-negative M-dimensional random vector $H{\left[i\right]}_{k}$. Figure 1D details the dimensions of each object. We use $V{\left[i\right]}_{j}$ to represent the jth feature in V[i], and $H_{k,j},H{\left[i\right]}_{k,j}$ to represent the jth feature in the kth source.

#### 2.1.2 Non-negative matrix factorization

Given a non-negative dataset V, the objective of NMF is to decompose the mixed signal into K sources, each represented by a constant component vector $H_{k}\in R_{\geq 0}^{M}$. The classic formulation from Lee and Seung (2000) is as an optimization problem (Eq. 2), where we look for two low-rank non-negative matrices s.t. their product is the closest to the observations, under some error function D. An equivalent formulation of the same problem is as a generative model for which we try to find the maximum-likelihood parameters under the assumptions of non-negativity and some noise model (Eq. 3, Lee and Seung 1999):

(2) $$\begin{array}{c} \hat{W},\hat{H}=\underset{W,H\geq 0}{\arg\min\;}D\left(V||R\left(W,H\right)\right)\\ \text{where}\;R\left(W,H\right)=W\cdot H\\ H\in R_{\geq 0}^{K\times M},\;W\in R_{\geq 0}^{N\times K}\\ \;⇕ \end{array}$$

 

(3) $$\begin{array}{c} \hat{W},\hat{H}=\underset{W,H\geq 0}{\arg\max\;}P\left(V|W,H\right)\\ \text{where}\;V{\left[i\right]}_{j}∼P_{\text{obs}}\left(R{\left[i\right]}_{j}\right)\\ R{\left[i\right]}_{j}=\sum_{k}W{\left[i\right]}_{k}\cdot H_{k,j} \end{array}$$

where $R{\left[i\right]}_{j}$ is the reconstruction of the mixed signal without noise. A graphical representation of this model is given in Supplementary Fig. S8. Specifically, we will focus on the KL-NMF model, where D is the KL-divergence error, and its equivalent generative formulation, where the observation probability $P_{\text{obs}}$ is a Poisson distribution — a common assumption in the case of biological sequencing data (Anders and Huber 2010).

This optimization problem is convex in W and H separately, but not together, thus we are only guaranteed to find local minima (Lee and Seung 2000). Many methods exist for approximating a solution. A simple and widely used method is an iterative algorithm called the *Multiplicative Update Rule* (Lee and Seung 2000) which is equivalent to block-wise gradient descent over W and H separately, while choosing a learning rate that keeps all elements of both matrices non-negative. In particular, the KL divergence is non-increasing under this multiplicative update rules and it is invariant under these updates if and only if W and H are at a stationary point of the divergence.

#### 2.1.3 Related works

A summary of notable previously published works is offered in Supplementary Fig. S7. We highlight here the main issues:

There are many extensions to NMF (see review in Wang and Zhang 2013), a common theme is the addition of some form of regularization, structure, or prior assumptions on the matrices H and W (i.e. assuming their values follow some distribution). Specifically, a Bayesian formulation of NMF was introduced in Schmidt *et al.* (2009), followed by many other formulations, including different choices of priors (e.g. Brouwer and Lio 2017) and hierarchical Bayesian models (e.g. Gopalan *et al.* 2015, Lu and Ye 2022). However, while these Bayesian extensions can integrate prior knowledge on the potential sources into the separation process, they still assume that constant components are shared between samples (even if these are integrated over; Brouwer and Lio 2017), and therefore they do not account for Type III variation in the source signals between samples; see Fig. 1.

Recently, Andrade Barbosa *et al.* (2021) suggested a probabilistic model which, similarly to VarNMF, accounts for Type III variation by modeling the components as distributions (in their case, log-normal distributions). However, these distributions are pre-learned, that is, estimated from direct observations of the sources. This approach can insert technical biases and fail in cases where there is no direct access to the sources, or if the sources that comprise the data are *a priori* unknown. For example, in cfChIP-seq data, one could theoretically assay many liver biopsies of different pathological states to estimate liver source variation. However, in practice, this approach is fraught with logistical and financial difficulties. For other tissue types (e.g. heart, brain) this is essentially impossible. Moreover, biopsy tissue can differ from the *in vivo* tissue, due to operation procedure, storage between operation and assay, and more. Here, we overcome this issue by estimating the sources’ distributions directly from the data.

Lastly, Rahmani *et al.* (2019), Wang *et al.* (2021) suggested tensor decomposition models, which learn the source distributions from data. They also estimate the per-sample sources signal H[i] for each sample i using a posterior calculation, similar to what we present below. However, their models assume Normal source distributions. As discussed in Andrade Barbosa *et al.* (2021), using this distribution can result in a biased fit of gene expression variability, compared to the non-negative alternatives considered. Furthermore, while they can restrict the mean of the distribution to achieve non-negative values, their posterior estimations of H[i] have no such restriction and are likely to include negative values to compensate for errors. It is therefore hard to interpret these estimations as per-sample sources signal. This, among other reasons detailed below, is why we focus instead on the non-negative Gamma distribution, which will result in non-negative mean and posterior estimations of the source signal, contributing to the interpretability of the results.

### 2.2 Method: VarNMF

#### 2.2.1 VarNMF model

We start with the probabilistic formulation of NMF from Eq. 3, and consider the possible inter-sample variation in source values (Type III variation). To model this variation, we take each component $H_{k}$ to be a random vector. That is, for each sample i, we have the latent components matrix:

(4) H[i]∈R≥0K×M s.t. H[i]k∼i.i.d by i pk

and the data distribution becomes:

(5) $$\begin{array}{c} V{\left[i\right]}_{j}∼\text{Poisson}\left(R{\left[i\right]}_{j}\right)\\ \;\text{where}\;R{\left[i\right]}_{j}=\sum_{k}W{\left[i\right]}_{k}\cdot H{\left[i\right]}_{k,j} \end{array}$$

This model is described in its graphical form in Supplementary Fig. S8. Importantly, the component signal is now a random vector that has its own instantiation for each sample, and is sampled from the source distribution $p_{k}$ with parameters $\theta_{H_{k}}$.

Now, given N independent observation vectors $V=\left(V\left[1\right],\ldots ,V\left[N\right]\right)\in R_{\geq 0}^{N\times M}$, we get the likelihood function $L_{\text{VarNMF}}(W,\theta_{H};V)=P\left(V|W,\theta_{H}\right)$, and look for the maximum-likelihood estimator (MLE) of $\theta =\left(W,\theta_{H}\right)$, that is, the proportion vectors $W\in R_{\geq 0}^{N\times K}$ and the source distributions’ parameters $\theta_{H}$ s.t.

(6) $$\hat{\theta }=\underset{W,\theta_{H}}{\arg\max\;}L_{\text{VarNMF}}(W,\theta_{H};V)$$

For simplicity, we assume that the different features in all components are independent. We also follow a common modeling choice for gene expression (Anders and Huber 2010) and assume that for each source k, the signal of feature j is distributed according to a Gamma distribution with its own parameters $\theta_{H_{k,j}}=\left(A_{k,j},B_{k,j}\right)$:

(7) $$\begin{array}{c} p_{k}(H{\left[i\right]}_{k})=\prod_{j}p_{k,j}(H{\left[i\right]}_{k,j})\\ =\prod_{j}p_{\text{Gamma}}\left(H{\left[i\right]}_{k,j};A_{k,j},B_{k,j}\right) \end{array}$$

#### 2.2.2 Likelihood function

The task defined by Eq. 6 is hard, as it involves integration over the latent matrix H. Specifically, computing the likelihood of a single observation requires a K-dimensional integral:

(8) $$\begin{array}{c} P\left(V{\left[i\right]}_{j}|\theta \right)=\int_{\vec{h}\in R_{\geq 0}^{K}}P\left(V{\left[i\right]}_{j}|H{\left[i\right]}_{:,j}=\vec{h},W\left[i\right]\right)\times \\ \times p\left(H{\left[i\right]}_{:,j}=\vec{h}|A_{:,j},B_{:,j}\right)d\vec{h} \end{array}$$

To partially alleviate this complexity, we separate the Poisson noise that differentiates the observation $V{\left[i\right]}_{j}$ from the reconstruction $R{\left[i\right]}_{j}$ (Eq. 5), to the noises originating from each of the sources. We do this by defining another set of latent variables Y, that represent the overall contribution of signal from each source to every sample, with its own Poisson noise:

(9) $$Y{\left[i\right]}_{k,j}∼\text{Poisson}\left(W{\left[i\right]}_{k}\cdot H{\left[i\right]}_{k,j}\right)$$

Using the fact that the sum of Poisson distributed random variables is a Poisson random variable (Lemma S4), we get the deterministic dependency:

(10) $$V{\left[i\right]}_{j}=\sum_{k}Y{\left[i\right]}_{k,j}$$

Now, given the values of Y, the components in H are independent of each other. Moreover, $P\left(Y{\left[i\right]}_{k,j};W\left[i\right],A_{k,j},B_{k,j}\right)$, which involves integration over $H{\left[i\right]}_{k,j}$, has a closed form solution — the commonly used negative Binomial distribution (Lemma S6). Thus, we can replace the K-dimensional integration in Eq. 8 with a K-dimensional summation that can be calculated using dynamic programming, and get the log-likelihood of the dataset V (Supplementary Section S2).

#### 2.2.3 Complete-data log-likelihood

While we can now theoretically optimize the likelihood by searching over the parameter space, this is infeasible in practice. Instead, we use the EM procedure (Supplementary Section S2 for full details). We start by examining the log-likelihood as though we observe the latent variables Y and H:

(11) $$\ell^{*}\left(\theta ;V,Y,H\right)\overset{\text{def}}{=}\;\text{log}\;p\left(V,Y,H|\theta \right)$$

which can be decomposed into three factors: log P(V | Y),  log P(Y|W,H), and log p(H|A,B). The first factor P(V | Y) is equal to 1 if Eq. 10 holds, and 0 otherwise. The second factor log P(Y | W,H) can be further decomposed for each sample i and source k into a separate log-likelihood function of the parameter $w=W{\left[i\right]}_{k}$, that accounts for the Poisson noise in $Y{\left[i\right]}_{k}$:

(12) $$\text{log}\;P\left(Y|W,H\right)=\sum_{i,k}\ell_{i,k}^{Y*}\left(W{\left[i\right]}_{k}\right)$$

 

(13) $$\ell_{i,k}^{Y*}\left(w\right)\overset{\text{def}}{=}\;\text{log}\;P\left(Y{\left[i\right]}_{k}|w,H{\left[i\right]}_{k}\right)$$

These likelihood functions have the following sufficient statistics:

(14) $$G{\left[i\right]}_{k}\overset{\text{def}}{=}\sum_{j}Y{\left[i\right]}_{k,j},T{\left[i\right]}_{k}\overset{\text{def}}{=}\sum_{j}H{\left[i\right]}_{k,j}$$

The last factor log p(H|A,B) represents the source distributions. Since we assumed independence between sources and between features, we can maximize the Gamma log-likelihood of each source k and feature j separately w.r.t. $a=A_{k,j}$ and $b=B_{k,j}$:

(15) $$\text{log}\;p\left(H|A,B\right)=\sum_{k,j}\ell_{k,j}^{H*}\left(A_{k,j},B_{k,j}\right)$$

 

(16) $$\ell_{k,j}^{H*}\left(a,b\right)\overset{\text{def}}{=}\;\text{log}\;p\left(H{\left[1\right]}_{k,j},\ldots ,H{\left[N\right]}_{k,j}|a,b\right)$$

using the sufficient statistics of the Gamma distribution:

(17) $$S^{0}\overset{\text{def}}{=}N,S_{k,j}^{1}\overset{\text{def}}{=}\sum_{i}H{\left[i\right]}_{k,j},S_{k,j}^{\;\text{log}\;}\overset{\text{def}}{=}\sum_{i}\;\text{log}\;H{\left[i\right]}_{k,j}$$

#### 2.2.4 EM procedure

Given a starting point $\theta^{\left(0\right)}=\left(W^{\left(0\right)},\theta_{H}^{\left(0\right)}\right)$, we apply the following Expectation (E-) and Maximization (M-) steps iteratively until convergence of the marginal log-likelihood $\ell_{\text{VarNMF}}\left(W,\theta_{H};V\right)=\text{log}\;P\left(V|W,\theta_{H}\right)$:

In the E-step, we calculate the expectation of sufficient statistics (the ESS) from Eq. 14 and Eq. 17 w.r.t. the posterior $p\left(Y,H|V,\theta^{\left(t\right)}\right)$. The full process is described in Supplementary Section S2, but essentially it is sufficient to calculate for each sample i and feature j the following probabilities:

(18) ∀k,d, p(V[i]j | Y[i]k,j=d;θ(t)) =p(∑l=kY[i]l,j=V[i]j−d;θ(t))

which can be achieved using the same dynamic programming procedure of the log-likelihood calculation.

In the M-step, we maximize the expectation of the complete-data log-likelihood:

(19) $$\theta^{\left(t+1\right)}=\underset{\theta }{\arg\max\;}E_{p\left(Y,H|V,\theta^{\left(t\right)}\right)}[\ell^{*}\left(\theta ;V,Y,H\right)]$$

From the linearity of expectation, we can find $\theta^{\left(t+1\right)}=\left(W,A,B\right)$ by separately maximizing $E\left[\ell_{i,k}^{Y*}\left(w\right)\right]$ and $E\left[\ell_{k,j}^{H*}\left(a,b\right)\right]$, using the ESS calculated in the E-step (see Supplementary Section S2).

#### 2.2.5 Convergence and implementation

Following the EM scheme, we assure convergence to a local maximum. In our case, the E-step is computationally demanding while the M-step is straightforward. As a starting point, we use the NMF solution of W and H (with a random start and the multiplicative update algorithm). We use the estimated H to initialize the mean of the Gamma distributions over H and initialize the variance s.t. the coefficient of variation is constant 1. We use a simple stopping criteria of T = 100 iterations for synthetic data and T = 250 for real data in the training stage, and run until convergence in the test stage (see below). An additional issue with both NMF and VarNMF solutions is that they are not identifiable, and many solutions will have the same log-likelihood. Therefore, to compare between solutions, we need to normalize. We expand on the normalization issue in Supplementary Section S2.

#### 2.2.6 Posterior profiles

Using the training data, we learn a distribution for each source, ${\hat{p}}_{k}=\text{Gamma}\left({\hat{A}}_{k},{\hat{B}}_{k}\right)$. The mean of these distributions can be interpreted similarly to the constant components extracted by NMF. However, under the VarNMF model, that accounts for Type III variation, each source k contributes some sample-specific signal $H{\left[i\right]}_{k}$ to sample i, weighted by $W{\left[i\right]}_{k}$. This sample-specific signal represents the isolated contribution of source k to sample i, and estimating it can help identify cases where the true contribution of the source to the particular sample is far from the mean, and thus indicate abnormal behavior. We estimate this signal using the expectation of the sample’s posterior source distribution:

(20) $$\hat{H}{\left[i\right]}_{k}=E\left[H{\left[i\right]}_{k}=h|V\left[i\right],{\hat{A}}_{k},{\hat{B}}_{k}\right]$$

which is calculated in the E-step (Supplementary Section S2). We call these estimations the *posterior profiles*.

## 3 Results

We start by applying VarNMF to synthetic data generated based on a cfChIP-seq dataset properties, with increasing variation in the sources signal. We compare the results with those of the NMF model (see Supplementary Section S3 for comparison with other methods). Next, we apply both algorithms to a cfChIP-seq dataset, to test real life performance. Finally, we conduct an additional performance analysis on pseudo-bulk RNA-seq data (Supplementary Section S5).

## 3.1 Synthetic data

To illustrate the capability of VarNMF for non-negative decomposition with source variation (Type III), we consider datasets of mixed samples with M = 100 features and K=1,…,10 sources. The sources are modeled as Gamma distributions with mean $\mu_{k,j}$ and variance $\sigma_{k,j}^{2}$ for the jth feature in the kth source. The means are generated by random permutations of an NMF solution trained on a real cfChIP-seq dataset. We control the Type III variation level by setting the coefficient of variation (CV) to a constant value, thus $\forall_{k,j},\;\sigma_{k,j}=\text{CV}\cdot \mu_{k,j}$. In each realization, we generate N = 100 samples from each source, starting with the sample-specific profiles (Lemma S2):

(21) $$\begin{array}{c} H{\left[i\right]}_{k,j}\overset{i.i.d.}{∼}\text{ Gamma}\left(A_{k,j},B_{k,j}\right)\\ \;\text{with}\;A_{k,j}=\frac{1}{{\text{CV}}^{2}},B_{k,j}=\frac{1}{{\text{CV}}^{2}\mu_{k,j}} \end{array}$$

We mix them using a weight matrix W, the rows of which are drawn independently from a symmetric Dirichlet(1) distribution, multiplied by a per-sample scaling factor λ[i]∼U([1,2]). We then sample an observation $V{\left[i\right]}_{j}$ from a Poisson with the mixture $R{\left[i\right]}_{j}=W{\left[i\right]}^{T}\cdot H{\left[i\right]}_{j}$ as its rate.

To evaluate VarNMF versus NMF, we examine their performance with the correct number of sources K (see Supplementary Section S3 for results with the wrong K). Since the number of parameters in VarNMF is higher than in NMF, we also apply NMF with $\tilde{K}$ sources, where $\tilde{K}$ is the minimal number to compensate for the difference in degrees of freedom.

Our goal is to extract accurate source distributions to better analyze new samples. Thus, we measure the generalization abilities of each model on a new dataset sampled from the same distribution. We create test datasets with $N_{\text{test}}=100$ and use a version of NMF and VarNMF to fit the new weight matrices $W_{\text{test}}$, while keeping the learned components\source distributions parameters constant (Supplementary Section S2).

**Figure 2.**
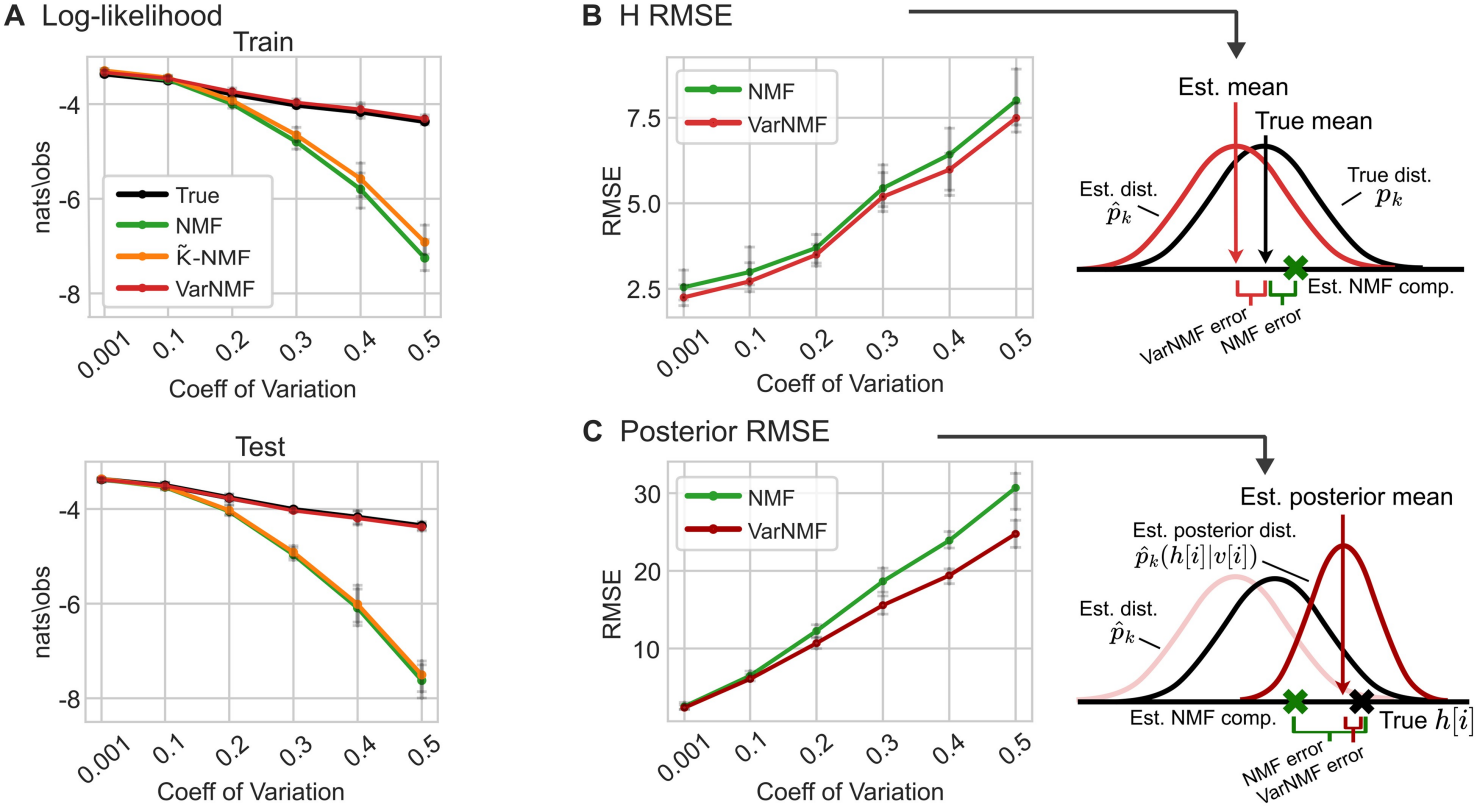
Decomposing synthetic data with K = 4 components: (A) Train and test log-likelihood of the ground truth parameters and three models—NMF, VarNMF, and $\tilde{K}$-NMF (NMF with higher degrees of freedom than VarNMF), versus the coefficient of variation of the dataset. The log-likelihood values are normalized to nats/observation. (B) Root mean square error (RMSE) of the mean of the distributions estimated by VarNMF (red on right panel) and the constant components estimated by NMF (green X) versus the mean of the ground truth distributions (black). (C) RMSE of the per-sample posterior profiles estimated by VarNMF (red) and the constant components estimated by NMF (green X) versus the ground truth H[i] (black X). (A, B, C) show results for 10 runs.

Applied to the synthetic datasets, the proposed VarNMF model achieves high log-likelihood for the train data, despite increasing CV (Fig. 2A and Supplementary Fig. S11). Although NMF has similar performance for low levels of Type III variation, its score drops sharply as these levels increase. This is not due to differences in the number of parameters, as $\tilde{K}$-NMF follows the same trend. Importantly, the test log-likelihood performances of all methods are similar to the train results, suggesting there is little overfitting. The results are also similar when increasing the number of samples N, the number of features M, and the observational noise λ (Supplementary Section S3). This suggests that VarNMF better captures the datasets distribution in the presence of high source variation. This advantage is clearer for smaller number of sources (Supplementary Fig. S15).

Next, we examine the learned components against the ground truth parameters. We observe that the means of the VarNMF distributions are closer to the ground truth means than the constant estimates of NMF (Fig. 2B and Supplementary Fig. S11). A similar improvement can be seen when comparing the ground truth per-sample contribution of source k, $H{\left[i\right]}_{k}$, to the VarNMF posterior profiles versus the NMF constant components (Fig. 2C and Supplementary Fig. S11). Overall, we conclude that for data with source variation, VarNMF learns a more accurate mean signal for the different sources, and allows for the estimation of per-sample source contributions via the posterior profiles.

## 3.2 Real data

We collected a dataset of cfChIP-seq samples from Sadeh *et al.* (2021) and Fialkoff *et al.* (2022). This data includes 80 plasma samples of healthy subjects, 139 samples of small-cell lung cancer (SCLC) patients, and 86 samples of colorectal cancer (CRC) patients (some patients were sampled at multiple time points). The two cancer cohorts represent different diseases, yet they are both solid tumors and are expected to have some common features. The cell-free literature reports on large variations in the fraction of tumor DNA in circulation (Zill *et al.* 2018). Thus, there is non-trivial heterogeneity in terms of the mixing proportions (Type II variation). There are also reports on molecular differences among cancers of the same type (Sadeh *et al.* 2021, Fialkoff *et al.* 2023), and therefore we expect to observe some variability in the signal of the component(s) representing each cancer type (Type III variation).

Out of this dataset, we select M = 7000 genes (Supplementary Section S4). Additionally, instead of training with the EM algorithm directly, we use a scheme that alternates optimization of W and A,B, to allow for parallel optimization of the Gamma distributions of the different features (Supplementary Section S2). Although this change does hinder the performance of VarNMF, it is necessary to allow runs with a large number of genes (Supplementary Section S3). Another adjustment for this data is for a non-specific background noise originating from the sequencing assay (Sadeh *et al.* 2021). This noise levels are estimated as part of the assay and we regard it as another source of the signal (i.e., another component), with pre-estimated contribution and signal. In particular, both NMF and VarNMF can incorporate this noise with minor changes to their training algorithms. We randomly divided the cohort into 185 training samples and 120 test samples (repeated 5 times). We train the different procedures on the training data with an increasing number of components K without any labels. We test the trained model using the same scheme as for synthetic data (Supplementary Section S2).

**Figure 3.**
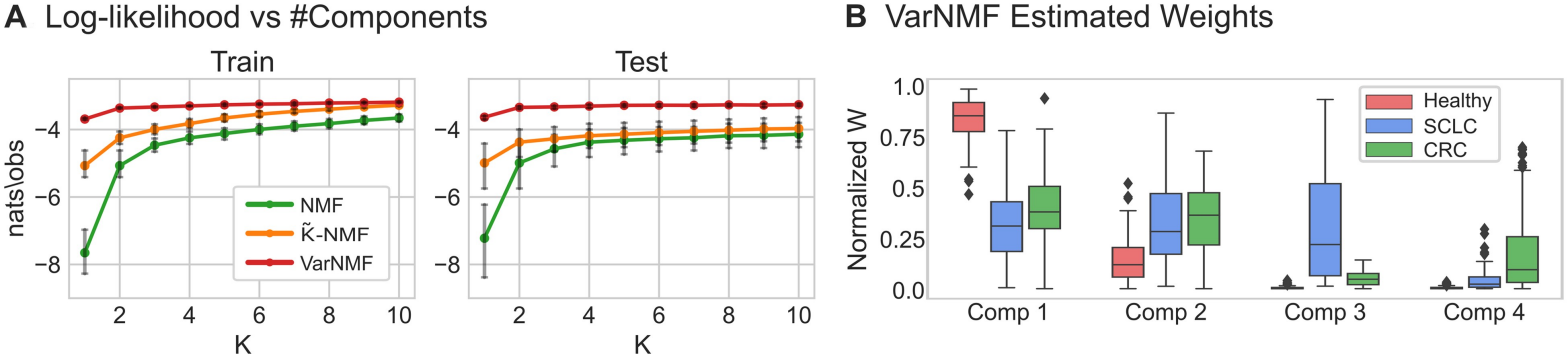
Decomposing cell-free ChIP-seq data: (A) Train and test log-likelihood curves for real cfChIP-seq dataset versus the number of components K used, for the three models and for 5 splits to train and test. The log-likelihood values are normalized to nats/observation. (B) The VarNMF estimated weights for each component in the K = 4 solution (train and test shown together), aggregated by sample sub-cohort (healthy, SCLC and CRC). Weights are normalized so that each sample has a total weight of 1.

We start by evaluating the ability of the different models to learn sources that generalize to new instances from the same cohort. Plotting the log-likelihood as a function of the number of components K (Fig. 3A), we observe that all models perform better on training data as K increases. However, there is a clear advantage for VarNMF on test data (∼1.2 nats/observation) that does not diminish for larger Ks. Consequently, we conclude that VarNMF is more effective at learning representations of the inherent structure in this data. Moreover, these results suggest the relevance of the source variation model for describing the underlying biology.

Next, we examine the biological relevance of the learned components in a specific solution. While there is no clear optimal K, and different values of K can potentially represent increasing levels of granularity, we choose the K = 4 solution as a representative example (see Supplementary Section S4 for analysis of other values of K). Examining the range of values of W for the three sub-cohorts (Fig. 3B), we see two cancer-specific components (that have non-zero weights mostly in one cancer type) — components #3 and #4 — and two shared “healthy” components (that are high in healthy samples but also appear in a range of weights in the cancer samples) — components #1 and #2. This is to be expected given that the cancer contributes only a part of the cell-free material in the plasma. Moreover, the values of W for the two cancer-specific components are correlated to supervised estimations of disease scores (r = 0.98 and 0.95, for $W_{3}$ and $W_{4}$ respectively, Supplementary Fig. S17).

An alternative way of interpreting the biological association of the components is to examine the mean contribution of a component to the value of each gene, $\mu_{k,j}=\frac{A_{k,j}}{B_{k,j}}$ or $H_{k,j}$. Specifically, we can choose genes that have high mean values in the component and low mean values in all other components (*differential genes*; Supplementary Section S4). We then test whether these genes are significantly over-represented in curated genes-lists associated with a specific tissue, cell-type or cell-line (Chen *et al.* 2013). Results for the first three components are similar between NMF and VarNMF. The first two components’ genes are strongly enriched for Platelets and Neutrophils, which are the two main sources of cell-free DNA in healthy samples (Moss *et al.* 2018, Sadeh *et al.* 2021). Component #2 is also enriched for Macrophages that differentiate from Monocytes which are also found in high concentrations in cell-free DNA from healthy samples. Component #3 (associated mainly with SCLC patients) is enriched for SCLC-derived cell-lines in both the NMF and VarNMF solutions. This indicates that this component indeed represents the tumor-derived cell-free DNA in the SCLC patients plasma. Component #4 (associated mainly with CRC patients) displays different associations between the two models. The NMF solution is enriched exclusively for colon-derived cell lines, aligning with the CRC patients diagnosis. On the other hand, the VarNMF solution is enriched for both colon and liver-derived cell lines. The latter enrichment may reflect the liver metastases or liver damage in many of the CRC patients in this cohort. We conclude that the mean of the components estimated by VarNMF captures the main expected contributors of cell-free DNA to the cohort samples. Importantly, the NMF solution yields a similar interpretation, and these findings are not exclusive to the VarNMF estimation.

**Figure 4.**
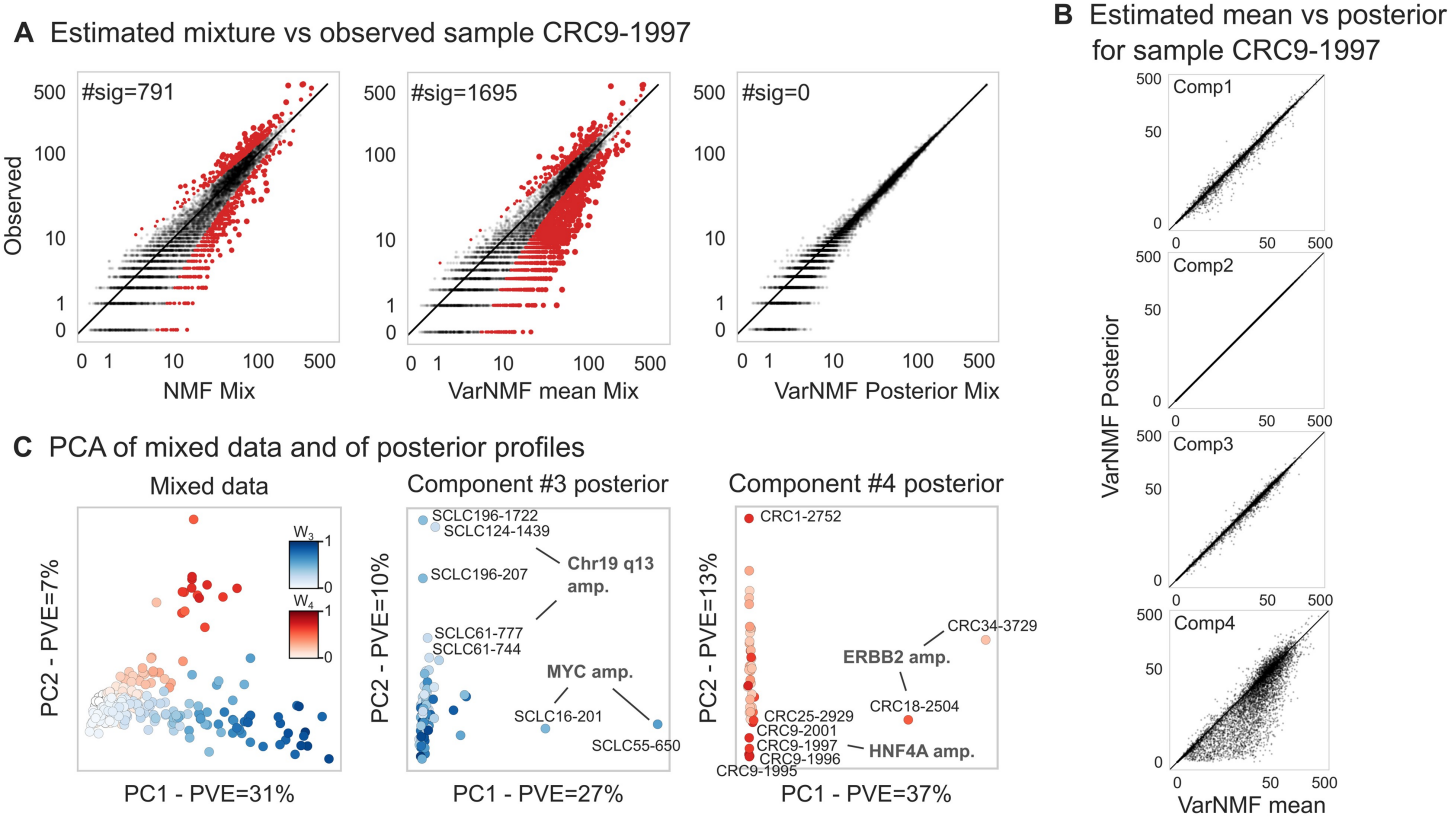
Reconstructing H from data with the K = 4 solution: (A) Reconstruction quality of a specific sample by NMF (left), VarNMF mean components (middle), and VarNMF posterior profiles (right). Each point is a gene, the *x*-axis shows the reconstructed value R and the *y*-axis shows the observed value V. Red points are ones that are significantly different, taking into account Poisson sampling noise (*q*-values corrected for false discovery rate < 0.05). (B) For the same example, the component mean vs. posterior profile per component. (C) PCA of the original mixed samples after normalization (Supplementary Section S4) (left) and of the component-wise posterior profiles (middle, right). Train and test samples are shown together. Only samples with weight >15% in the relevant components are shown.

To illustrate the unique features of VarNMF, we examine a specific sample of a CRC patient (Fig. 4A, see Supplementary Fig. S19 for more examples). Mixing the NMF constant components according to the weights learned for this sample, results in a reconstruction that significantly diverges from the observed signal in hundreds of genes. Similarly, mixing the means of the source distributions learned by VarNMF according to the VarNMF weights for the sample, also results in many unexplained genes, even more than for the NMF reconstruction. However, when using the sample-specific per-component posterior profiles estimated by VarNMF in the reconstruction, the observed signal is fully explained. Thus, while much of the variation between samples can be accounted for by the mixing proportions, there is a non-trivial additional layer of variability due to Type III variation — variation in signals within components. Examining these posterior profiles for the sample (Fig. 4B), we note that while in the first three components, the posterior signal is close to the component mean, the fourth CRC-associated component exhibits a sample-specific signal. This suggests that most of the discrepancies originate from the CRC source, i.e. that the disease-specific features explain most of the previously unexplained signals in the sample. These behaviors are repeated in most samples in both the train and test datasets (see Supplementary Fig. S20 for more examples).

Looking at this phenomena more generally (Fig. 4C), the main directions of variation in the mixed samples are the percentage of disease — Type II variation (Pearson correlation of PC1 and $W_{3}=0.89$; of PC2 and $W_{4}=0.84$). In contrast, the main directions of variation in the posterior profiles for disease-associated sources are no longer linked to the degree of disease, but rather to unique characteristics of sub-populations of patients — Type III variation. As a result, these estimations allow us to separate sample-specific disease behaviors from the mixed samples, and examine inter-cancer variability. For example, in the posterior profiles of the CRC-associated component (component #4), the first two PCs separate two sub-populations from the main population of patients. These sub-populations have two different known genomic amplifications (ERBB2 with PVE = 37% and HNF4A with PVE = 13%; Sadeh *et al.* 2021). Similarly, in the posterior profiles of the SCLC-associated component (component #3), PC1 is associated with MYC amplification (PVE = 27%) and PC2-5 with other specific amplifications (PVE = 10%–3%). Genomic amplification results in increased copy-number of a chromosomal region, thereby amplifying the expression of one or more oncogenes that contribute to the increased fitness of cells with the amplification. Therefore, identifying these amplifications can be relevant to prognosis, treatment planning, etc.

**Figure 5.**
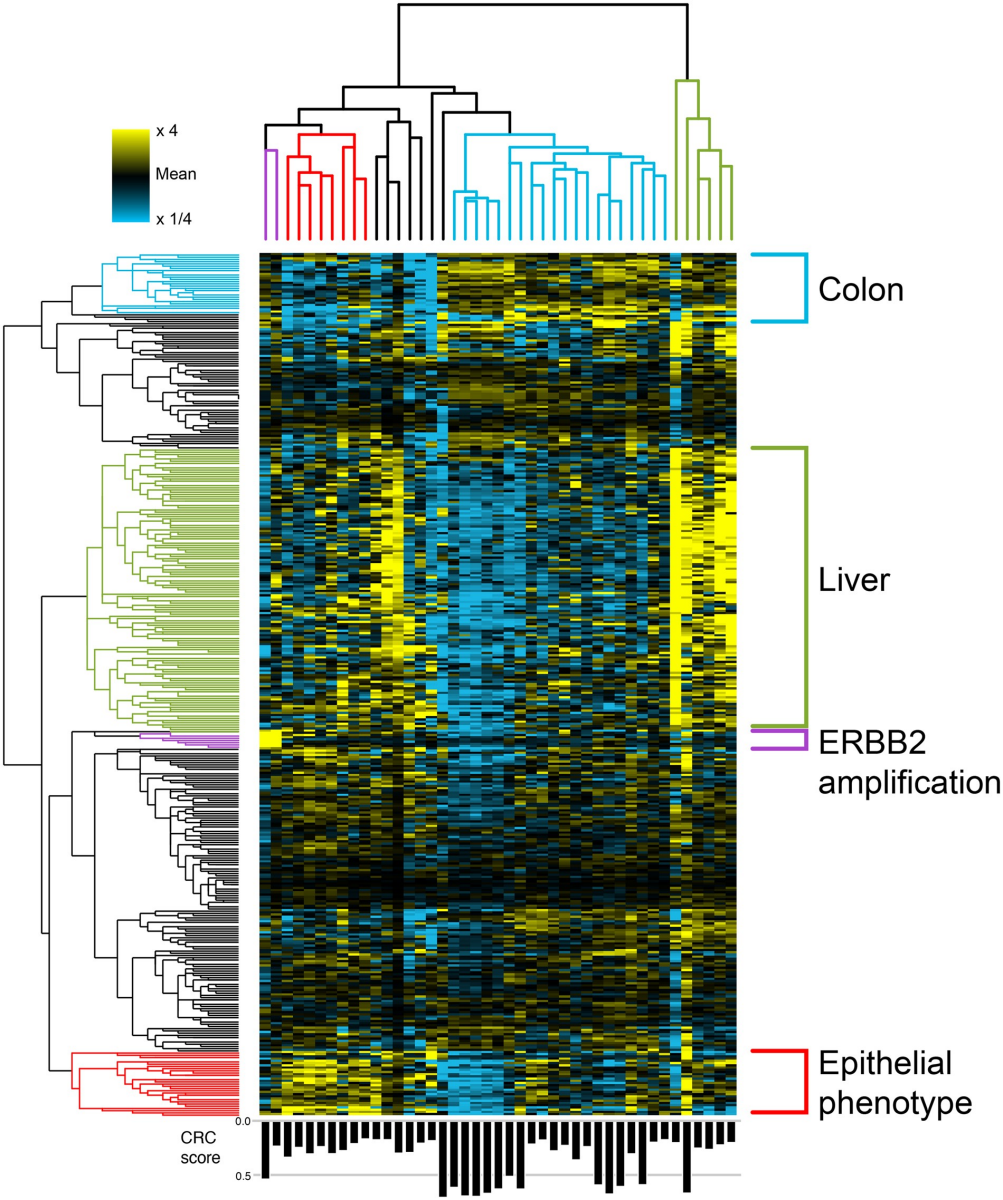
Clustering of the CRC-related posterior profiles (relative to the mean signal of each gene, with color saturation at 4-fold increase/decrease) in 383 genes that are significantly higher in the CRC-related component mean compared to the other components, and additional six genes that are in the ERBB2 amplification. Four sample — gene clusters were identified: ERBB2 amplification (purple), samples with an epithelial phenotype (red), samples with specific colon behavior (light blue) and samples with high liver signal (green). Only samples with weight >15% in the CRC-related component were considered. Samples weight in the CRC-related component ($W_{4}$) are indicated in the bar plot in the lower panel.

Beyond amplifications, we can also examine more complex patterns of variation. Focusing on the CRC-related posterior profiles — our estimation of the pure tumor behavior in the patients, before its dilution with other sources in the plasma — we obtain a complex cluster map (Fig. 5). We identify four main pairs of sample-gene clusters, where the relevant genes are elevated in the associated samples. They match biological aspects of the patients and cancer, such as ERBB2 genomic amplification, but also functional phenotype of the tumor, and increased liver damage. Importantly, the clustering is not due to the amount of tumor contribution (Type II variation; lower panel of Fig. 5). Ultimately, using this estimation for the different samples and components decomposed by VarNMF, allows us to explore the Type III variation in the data.

## 4 Discussion

Here, we presented VarNMF, a model for decomposition of non-negative data into source distributions and mixing proportions, in a way that tackles variation in source values between samples (Type III variation). We evaluated VarNMF on synthetic data and found that in the presence of source variation, it better generalizes to unseen samples and offers a more accurate representation of the data distribution, compared to NMF. We then evaluated VarNMF on pseudo-bulk RNA-seq data, where real-life profiles were mixed to generate bulk samples with known mixing proportions. We further applied VarNMF to real-life cfChIP-seq data, illustrating its capacity to decompose real-world data into biologically relevant source distributions that accurately represent the entire population. Additionally, VarNMF estimates the sample-specific posterior profile of a source, which reflects the source contribution to a specific sample and can indicate patient-specific disease behavior. This potentially allows for direct access to disease behavior in patients across time and following treatment in a non-invasive manner.

More generally, we believe that source variation is prevalent in many scenarios where NMF is applied to biological data. In bulk samples, for example, contributing cells of some cell type can deviate from the stereotypical profile of their type. While in theory, one can compensate for source variation by adding multiple related constant components that span the variation (e.g. inflamed hepatocytes vs. healthy hepatocytes), this solution is less robust and harder to interpret. Moreover, the distinction between mixing proportions (Type II variation) and source values (Type III) allows estimation of the unique profile in each sample. As we showed, such estimation uncovers additional biologically meaningful patterns in the data.

We presented VarNMF in the simplest form, and many improvements can be introduced. For example, while the alternating EM procedure allows for scaling to large datasets, it remains computationally expensive, and further efforts may be taken to speed up training. Learning a prior distribution over W can provide another boost in generalization. The model of $p(H_{k})$ is simple and does not capture dependencies between features, although non-trivial dependencies are expected in genomic data. In principle, the modeling framework presented here can be extended to include these modeling changes.

## 5 Conclusion

From a broader perspective, the approach we presented provides a framework for learning about the distributions of complex latent sources without observing them directly, a recurring challenge when attempting to study *in vivo* molecular states from compound observations. This raises interesting questions regarding the limits on generalizing such models from mixed observations.

## Supplementary Material

btae758_Supplementary_Data

## Data Availability

cfChIP data are based on Sadeh *et al.* (2021) (publicly available data) and Fialkoff *et al.* (2022) (provided through authors).
