# Supplementary material for: VarNMF: non-negative probabilistic factorization with source variation
Source: Bioinformatics. 2024 Dec 28;41(1):btae758. doi: 10.1093/bioinformatics/btae758 (PMC11979754; doi:10.1093/bioinformatics/btae758)
Supplement: btae758_Supplementary_Data [file btae758_supplementary_data.zip › 14ff3_bioinformatics_submission___supp.pdf]

Supplementary Material

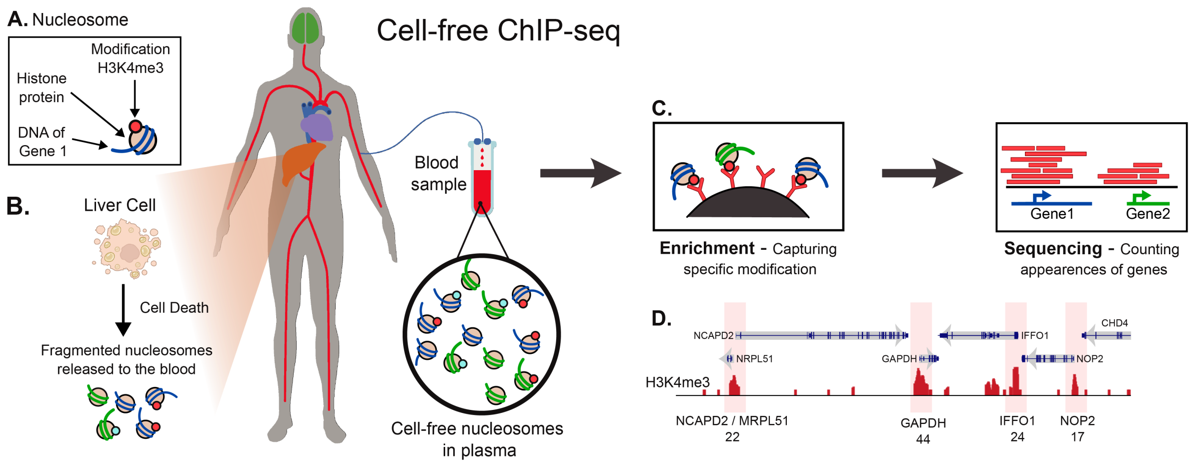

Fig. 6. cfChIP concept - A) Genomic DNA in the nucleus is packaged into nucleosome complexes made of DNA wrapped around histone proteins. The histone protein at each nucleosome can be modified in a way that is tightly coordinated with gene activity at that position [Soares et al., 2017]. B) Upon cell-death, the genome is fragmented and nucleosomes are released into the circulation as cell-free nucleosomes that retain their modifications [Aucamp et al., 2018]. C) cfChIP-seq [Sadeh et al., 2021] uses immunoprecipitation to capture modified nucleosomes from plasma, and then sequence the DNA fragments bound to these nucleosomes. By mapping these sequences to the genome, we can associate them with genes. Thus, similar to RNA-seq data, this assay provides a quantitative signal of activity associated with each gene. This signal reflects the aggregate contribution of all cells that released modified nucleosomes into the circulation, and thus if we could break it into individual components, we would be able to report on each sub-population of cells (e.g., tumor cells, immune cells). D) Illustration of how cfChIP-seq results are quantified. For each gene we use a pre-defined promoter region (pink box), and we count the number of fragments whose center lies within the region. The resulting vector of counts per gene is processed to estimate background and possible normalization [Sadeh et al., 2021].

1. Comparison of alternative models

There are multiple differences between the various related approaches in the literature. In Figure 7, we compare multiple methods along with what we believe are the salient aspects. In addition, in Figure 8 we compare the probabilistic models of NMF, Bayesian NMF, and VarNMF. While these are similar, there are crucial differences in the scope of the plates involving H.

<table>
<tr><th>Method</th><th>Assumes biological variation between samples in component signals</th><th>Uses prior knowledge (e.g. single-cell data)</th><th>Estimates the component proportions W</th><th>Calculates per-sample component signal</th><th>Non-negativity assumption</th></tr>
<tr><td>NMF</td><td rowspan="2">No. Variability between samples is only due to component composition and technical noise</td><td rowspan="2">Possible, but not necessary</td><td rowspan="2">Yes. Estimates W without prior knowledge</td><td rowspan="2">No</td><td rowspan="2">Yes</td></tr>
<tr><td>Bayesian NMF</td></tr>
<tr><td>CIBERSORTx</td><td>Yes. Allows both group-mode estimation and “high-resolution” per-sample estimation</td><td>Yes. To construct signature matrix</td><td rowspan="3">Yes. Estimates W given prior knowledge of cell-types in the data</td><td>Yes. Uses a heuristic calculation based on differential genes</td><td>Yes</td></tr>
<tr><td>BayesPrism</td><td>Partially. Only accounts for differences in cell-state composition in each cell type</td><td>Yes. To construct cell-state profiles</td><td rowspan="4">Yes. Uses posterior estimation</td><td>Yes</td></tr>
<tr><td>BLADE</td><td rowspan="3">Yes. Each cell-type is modeled as a distribution</td><td>Yes. To estimate cell-type distributions</td><td>Yes. Assumes log-normal cell-type distributions</td></tr>
<tr><td>bMIND</td><td>Yes. To estimate priors on cell-type distributions</td><td rowspan="2">No. Assumed known or pre-estimated W</td><td rowspan="2">No. Assumes Normal cell-type distributions -> No non-negativity assumption on the per-sample cell-type values</td></tr>
<tr><td>TCA</td><td>No</td></tr>
<tr><td>debCAM + swCAM</td><td>Yes. Allows deviation from the mean signal of each cell-type</td><td>No</td><td>Can estimate W using debCAM</td><td>Yes. Approximates the deviation of each sample as a sparse matrix</td><td>No. Assumes that the sample-specific signal is a positive semi-definite matrix</td></tr>
<tr><td>VarNMF</td><td>Yes. Each cell-type is modeled as a distribution</td><td>Possible, but not necessary</td><td>Yes. Estimates W without prior knowledge</td><td>Yes. Uses posterior estimation</td><td>Yes. Assumes Gamma component distributions</td></tr>
</table>

Fig. 7. Related works: Table highlighting the main features of VarNMF and previous works in the field. Red boxes indicate main differences. NMF: Lee and Seung [2000], Bayesian NMF: Schmidt et al. [2009], CIBERSORTx: Newman et al. [2019], BayesPrism: Chu et al. [2022], BLADE: Andrade Barbosa et al. [2021], bMIND: Wang et al. [2021], TCA: Rahmani et al. [2019], debCAM: Chen et al. [2020], swCAM: Chen et al. [2022]

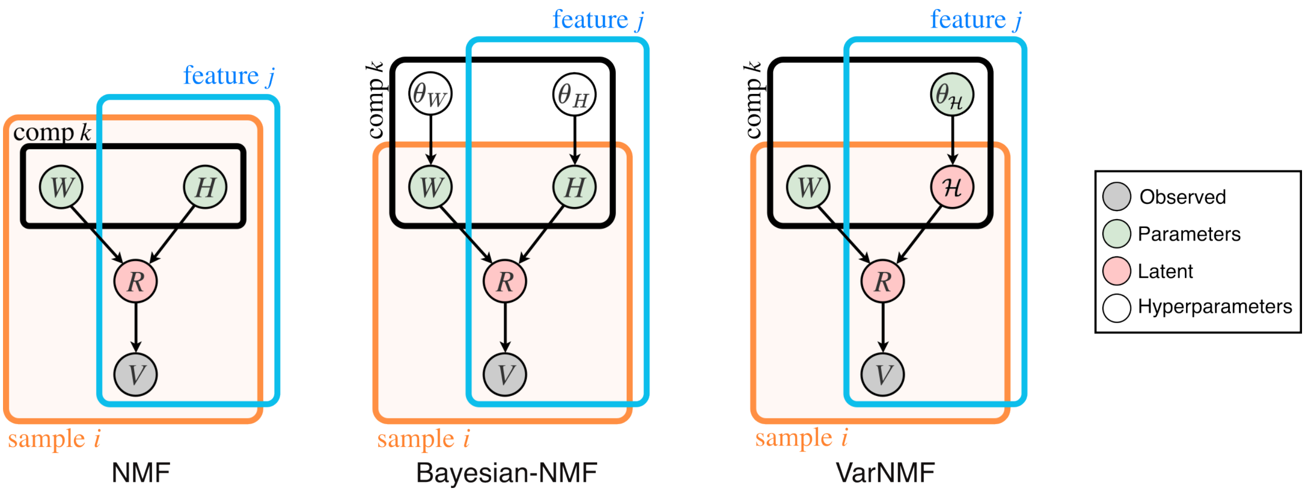

Fig. 8. Graphical plate representation [Koller and Friedman, 2009] for the NMF, Bayesian NMF and VarNMF models. Latent variables are ones that are integrated over in the model. Parameters are point estimates either by Maximum Likelihood or Maximum a Posteriori. Hyperparameters are pre-learned.

2. EM details

Distributions definitions and properties

We use the following parametrizations of the Poisson, Gamma and NB distributions:

Definition 1 (Poisson Parametrization) We say that $Y \sim \text{Poisson}(\lambda)$ if

$$P(Y = k) = \frac{1}{k!} \cdot \lambda^k \cdot e^{-\lambda}$$

Lemma 1 (Poisson Properties) For $Y \sim \text{Poisson}(\lambda)$,

$$\mathbb{E}[Y] = \lambda, \quad \text{Var}[Y] = \lambda, \quad \text{CV}[Y] = 1/\sqrt{\lambda}$$

Definition 2 (Gamma Parametrization) We say that $X \sim \text{Gamma}(\alpha, \beta)$ if

$$p(X = x) = \frac{\beta^\alpha}{\Gamma(\alpha)} \cdot x^{\alpha-1} \cdot e^{-\beta x}$$

Lemma 2 (Gamma Properties)

1. For $X \sim \text{Gamma}(\alpha, \beta)$,

$$\mathbb{E}[X] = \frac{\alpha}{\beta}, \quad \text{Var}[X] = \frac{\alpha}{\beta^2}, \quad \text{CV}[X] = 1/\sqrt{\alpha}$$

2. If

$$\mathbb{E}[X] = \mu, \quad \text{Var}(X) = \sigma^2$$

then

$$X \sim \text{Gamma}\left(\alpha = \frac{\mu^2}{\sigma^2}, \beta = \frac{\mu}{\sigma^2}\right)$$

3. If

$$\mathbb{E}[X] = \mu, \quad \text{CV}[X] = \text{cv}$$

then

$$X \sim \text{Gamma}\left(\alpha = \frac{1}{\text{cv}^2}, \beta = \frac{1}{\mu \cdot \text{cv}^2}\right)$$

Definition 3 (Negative Binomial Parametrization) We say that $X \sim \text{NB}(r, p)$ if

$$P(X = k) = \binom{k + r - 1}{k} \cdot p^k \cdot (1 - p)^r$$

Lemma 3 (Negative Binomial Properties) For $X \sim \text{NB}(r, p)$,

$$\mathbb{E}[X] = \frac{pr}{1-p}, \quad \text{Var}[X] = \frac{pr}{(1-p)^2}, \quad \text{CV}[X] = 1/\sqrt{pr}$$

We state lemmas concerning properties of the Gamma and Poisson distributions [Wackerly et al., 2014]:

Lemma 4 If $X_k \sim \text{Poisson}(\lambda_k)$ for $k = 1, \ldots, K$, then $X = \sum_{k=1}^K X_k \sim \text{Poisson}\left(\sum_{k=1}^K \lambda_k\right)$.

Lemma 5 If $X \sim \text{Gamma}(\alpha, \beta), \gamma > 0$ then $\gamma \cdot X \sim \text{Gamma}\left(\alpha, \frac{\beta}{\gamma}\right)$.

Lemma 6 If $Y \mid X \sim \text{Poisson}(X)$ and $X \sim \text{Gamma}(\alpha, \beta)$ then $Y \sim \text{NB}\left(\alpha, \frac{1}{1+\beta}\right)$.

Lemma 7 If $Y \mid X \sim \text{Poisson}(\gamma X), \gamma > 0$ and $X \sim \text{Gamma}(\alpha, \beta)$ then

$$(X|Y = t) \sim \text{Gamma}(\alpha + t, \beta + \gamma)$$

Lemma 8 If $X \sim \text{Gamma}(\alpha, \beta)$ then $\mathbb{E}[\log(X)] = \psi(\alpha) - \log\beta$, where ψ is the digamma function.

Log-likelihood

First, we present a scheme that allows us to calculate the log-likelihood of a set of parameters $\theta = W, A, B$. We can calculate the log-likelihood for each observation separately:

$$\ell(\theta; V) \stackrel{\text{def}}{=} \log \sum_{i,j} p\left(V[i]_j \mid W[i], A_{:,j}, B_{:,j}\right) \tag{23}$$

To simplify notation, we set i, j and define

$$v = V[i]_j, y = \mathcal{Y}[i]_{:,j}, h = \mathcal{H}[i]_{:,j}, a = A_{:,j}, b = B_{:,j}, w = W[i], \theta = (w, a, b) \tag{24}$$

and look to calculate $\log p(v \mid \theta)$. As mentioned above, with the addition of the random variables Y to the model, this require a K-dimensional summation, and will be done using dynamic programming.

It is sufficient to calculate the joint distribution of (y_k, v) for some k, since:

$$p(v \mid \theta) = \sum_{d=0}^{v} p(y_k = d, v \mid \theta) \tag{25}$$

Moreover, we can write this joint as

$$p(y_k = d, v \mid \theta) = p(y_k = d \mid \theta) \cdot p(v \mid y_k = d, \theta) \tag{26}$$

The first factor can be directly calculated using the following lemma:

Lemma 9

$$\begin{cases} (y_k \mid w_k, h_k) \sim \text{Poisson}(w_k \cdot h_k) \\ (h_k \mid a_k, b_k) \sim \text{Gamma}(a_k, b_k) \end{cases} \Rightarrow \quad (y_k \mid w_k, a_k, b_k) \sim \text{NB}\left(a_k, \frac{w_k}{w_k + b_k}\right)$$

Proof From Lemma 5,

$$(h_k \mid a_k, b_k) \sim \text{Gamma}(a_k, b_k) \Rightarrow \quad (w_k \cdot h_k \mid w_k, a_k, b_k) \sim \text{Gamma}\left(a_k, \frac{b_k}{w_k}\right)$$

and from Lemma 6 we get

$$\begin{cases} (y_k \mid w_k, h_k) \sim \text{Poisson}(w_k \cdot h_k) \\ (w_k \cdot h_k \mid w_k, a_k, b_k) \sim \text{Gamma}\left(a_k, \frac{b_k}{w_k}\right) \end{cases}$$

and therefore

$$(y_k \mid w_k, a_k, b_k) \sim \text{NB}\left(a_k, \frac{1}{1 + \frac{b_k}{w_k}}\right) = \text{NB}\left(a_k, \frac{w_k}{w_k + b_k}\right)$$

□

As for the second factor, $v = \sum_k y_k$ and v is discrete, therefore

$$\forall_{k,d}, \; p(v \mid y_k = d, \theta) = p\left(\sum_{l \neq k} y_l = v - d \mid \theta\right) \tag{27}$$

and that can be calculated using dynamic programming:

For simplicity, we denote $p = p_\theta$ and $p_k = \mathrm{NB}\left(a_k, \frac{w_k}{w_k+b_k}\right)$ (the distribution of y_k). We define two random variables:

$$X_s \stackrel{\text{def}}{=} \sum_{l=1}^{s} y_l \qquad\qquad Z_s \stackrel{\text{def}}{=} \sum_{l=s+1}^{K} y_l \tag{28}$$

and two tables

$$F[s,n] \stackrel{\text{def}}{=} p\left(X_s = n\right), \quad s = 1, \ldots, K-1, \;\; n = 0, \ldots, v \tag{29}$$

$$B[s,n] \stackrel{\text{def}}{=} p\left(Z_s = n\right), \quad s = 1, \ldots, K-1, \;\; n = 0, \ldots, v \tag{30}$$

Using the law of total probability, we get the two following recursive formulas:

$$F[s,n] = \sum_{d=0}^{n} F[s-1,d] \cdot p_s(n-d) \tag{31}$$

$$B[s,n] = \sum_{d=0}^{n} B[s+1,d] \cdot p_{s+1}(n-d) \tag{32}$$

Now we have two dynamic programming tasks:

1. The Forward task of filling F by columns, from the initialization:

$$\forall_{0\le n\le v}, \;\; F[1,n] = p_1(n)$$

 and forward according to Eq. 31.
2. The Backward task of filling B by columns, with initial values for the last column:

$$\forall_{0\le n\le v}, \;\; B[K-1,n] = p_K(n)$$

 and going backward with Eq. 32.

Finally, to find the required probability we use these two tables to fill a new table

$$P[k,d] \stackrel{\text{def}}{=} p\left(v \mid y_k = d\right), \quad k = 1, \ldots, K, \;\; d = 0, \ldots, v \tag{33}$$

using the recursive formula

$$\begin{aligned} \forall_{1<k<K}, \forall_d, \quad P[k,d] &= p\left(\sum_{l\neq k} y_l = v-d\right) \\ &= p\left(\sum_{l=0}^{k-1} y_l + \sum_{l=k+1}^{K} y_l = v-d\right) \\ &= p\left(X_{k-1} + Z_k = v-d\right) \\ &= \sum_{n=0}^{v-d} p\left(Z_k = v-d-n\right)\cdot p\left(X_{k-1} = n\right) \\ &= \sum_{n=0}^{v-d} B[k, v-d-n] \cdot F[k-1,n] \end{aligned} \tag{34}$$

and the initial condition

$$\forall_d, \;\; P[K,d] = F[K-1, v-d] \tag{35}$$

Complete-data log-likelihood

To apply the EM procedure to our model, we need to calculate the expectations of the following sufficient statistics to get the ESS in the E-step:

$$G[i]_k = \sum_j \mathcal{Y}[i]_{k,j}, \qquad\qquad T[i]_k = \sum_j \mathcal{H}[i]_{k,j} \tag{36}$$

$$S^0 = N, \qquad S^1_{k,j} = \sum_i \mathcal{H}[i]_{k,j}, \qquad S^{\log}_{k,j} = \sum_i \log \mathcal{H}[i]_{k,j} \tag{37}$$

and to maximize the expectation of the complete-data log-likelihood in the M-step:

$$\theta^{(t+1)} = \arg\max_\theta \mathbb{E}_{p\left(\mathcal{Y},\mathcal{H}\mid V,\theta^{(t)}\right)}\left[\ell^*\left(\theta; V, \mathcal{Y}, \mathcal{H}\right)\right] \tag{38}$$

Here, we provide the full mathematical details of these steps:

M - step
As mentioned above, the maximization of Eq. 38 can be achieved by separately maximizing the log-likelihood functions $\ell_{i,k}^{\mathcal{Y}*}$ and $\ell_{k,j}^{\mathcal{H}*}$, only with the ESS calculated in the E-step replacing the actual sufficient statistics.

Specifically, $\mathcal{Y}[i]_{k,j} \sim \text{Poisson}\left(W[i]_k \cdot \mathcal{H}[i]_{k,j}\right)$, therefore for each $w = W[i]_k$,

$$\begin{aligned}\ell_{i,k}^{\mathcal{Y}*}(w) &\stackrel{\text{def}}{=} \log P\left(\mathcal{Y}[i]_k \mid w, \mathcal{H}[i]_k\right) \\ &= \text{const} + G[i]_k \cdot \log w - T[i]_k \cdot w\end{aligned} \tag{39}$$

Additionally, $\mathcal{H}[i]_{k,j} \sim \text{Gamma}\left(A_{k,j}, B_{k,j}\right)$ and thus for each $a = A_{k,j}, b = B_{k,j}$,

$$\begin{aligned}\ell_{k,j}^{\mathcal{H}*}(a,b) &\stackrel{\text{def}}{=} \log p\left(\mathcal{H}[1]_{k,j}, ..., \mathcal{H}[N]_{k,j} \mid a, b\right) \\ &= [a \log b - \log \Gamma(a)] \cdot S^0 - b \cdot S^1_{k,j} + (a-1) \cdot S^{\log}_{k,j}\end{aligned} \tag{40}$$

Now, given the ESS, we maximize both functions separately. Set i, j, k, and

$$G = G[i]_{k,j}, \qquad T = T[i]_{k,j}, \qquad S^1 = S^1_{k,j}, \qquad S^{\log} = S^{\log}_{k,j} \tag{41}$$

Then, differentiating each function and setting the gradient to 0,

1. For $\ell^{\mathcal{Y}*}$ we get that $\hat{w} = \frac{G}{T}$.
2. For $\ell^{\mathcal{H}*}$, we get the following system,

$$\begin{cases}\psi(a) - \frac{S^{\log}}{S^0} - \log a + \log \frac{S^1}{S^0} = 0 \\ b = a \cdot \frac{S^0}{S^1}\end{cases}$$

where $\psi(x) = \frac{(\Gamma(x))'}{\Gamma(x)}$ is the digamma function. The first equation can be solved by finding a root using the Newton-Raphson algorithm.

E - step
As described above, given $\theta^{(t)} = W, A, B$, we are required to calculate the expectation of the sufficient statistics from Eq. 36 and Eq. 37. To simplify notation, and as this process is done separately for each feature j in each sample i, we set i, j and define

$$v = V[i]_j, y = \mathcal{Y}[i]_{:,j}, h = \mathcal{H}[i]_{:,j}, a = A_{:,j}, b = B_{:,j}, w = W[i], \theta^{(t)} = (w, a, b) \tag{42}$$

From linearity of expectation, it is enough to calculate for each k,

$$\text{(i)}\ \mathbb{E}\left[y_k \mid v, \theta^{(t)}\right] \qquad \text{(ii)}\ \mathbb{E}\left[h_k \mid v, \theta^{(t)}\right] \qquad \text{(iii)}\ \mathbb{E}\left[\log h_k \mid v, \theta^{(t)}\right] \tag{43}$$

We start by calculating the posterior distribution of y_k, $p(y_k = d \mid v, \theta)$ for each $0 \le d \le v$. This can be done using the joint probability $p(y_k = d, v \mid \theta)$ calculated with the dynamic programming scheme from 9.2, and the fact that

$$\begin{aligned}p(y_k = d \mid v, \theta) &= \frac{p(y_k = d, v \mid \theta)}{p(v \mid \theta)} \\ &= \frac{p(y_k = d, v \mid \theta)}{\sum_{l=0}^{v} p(y_k = l, v \mid \theta)}\end{aligned} \tag{44}$$

Given the posteriors, $\{p(y_k = d \mid v, \theta)\}_{d=1}^{v}$, we can now calculate:

1. $\mathbb{E}[y_k \mid v, \theta] = \sum_{d=0}^{v} d \cdot p(y_k = d \mid v, \theta)$.
2. $\mathbb{E}[h_k \mid v, \theta] = \frac{a_k + \mathbb{E}[y_k \mid v, \theta]}{b_k + w_k}$ (Lemma 7).
3. $\mathbb{E}[\log h_k \mid v, \theta] = -\log(b_k + w_k) + \sum_{d=0}^{v} p(y_k = d \mid v, \theta) \cdot \psi(a_k + d)$ (Lemmas 7, 8).

Calculating the posterior profiles

Given a source distribution (for some feature j) $p_{k,j} = \text{Gamma}\left(A_{k,j}, B_{k,j}\right)$ estimated from training data, we want to estimate the source contribution to a particular sample i, $\mathcal{H}[i]_{k,j}$. We use the expectation of the posterior distribution of $\mathcal{H}$:

$$\hat{p}[i]_{k,j}(h) = p\left(\mathcal{H}[i]_{k,j} = h \mid V[i], A_{k,j}, B_{k,j}\right) \tag{45}$$

Specifically, we use $\hat{\mathcal{H}}[i]_{k,j} = \mathbb{E}\left[\mathcal{H}[i]_{k,j} \mid V[i], A_{k,j}, B_{k,j}\right]$ which we calculated in the E-step as the sample-specific posterior profile (Eq. 43(ii)).

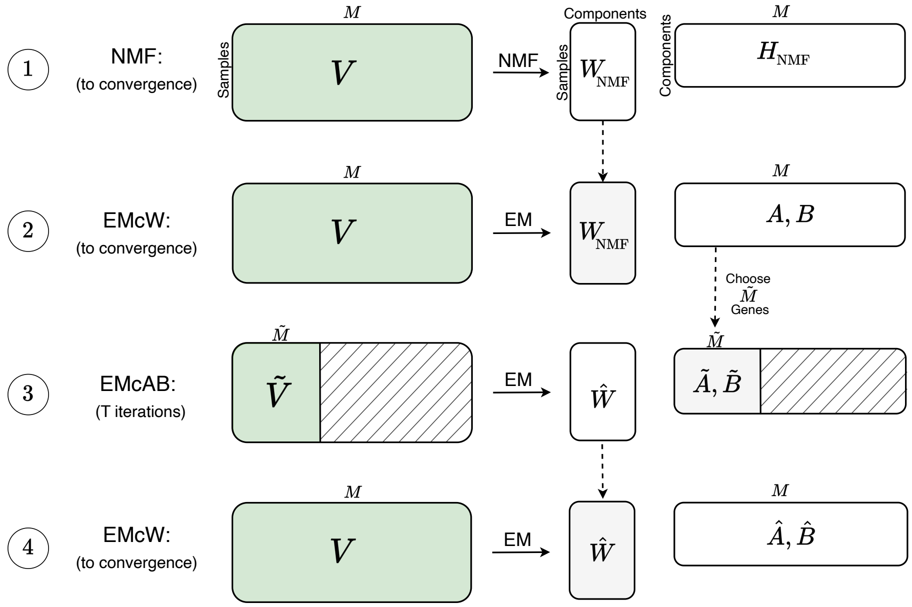

Fig. 9. The alternating EM procedure attempts to find an optimal solution for the VarNMF model while avoiding high computational costs. It uses the NMF solution as a starting point (step 1), and applies EM over one of the parameter matrices W or (A, B) at a time (steps 2 and 3). This process is then repeated once in step 4 for W, and can be applied iteratively. Green indicates a data matrix (or a subset of one) and grey indicates a fixed parameter matrix (that results from the previous step). $T = 250$ for all real-data runs.

Alternating EM

As mentioned above, the E-step of the presented EM procedure is computationally heavy, and cannot be applied on a real dataset with a typical number of a few thousand features (genes). There are many possible solutions to this problem in literature. Here, we decided to use an alternating version of EM [Neal and Hinton, 1998], which also alternates between optimizing W and A,B (similar to the NMF multiplicative update rule). The algorithm steps are detailed in Fig. 9.

We start in step 1 with an NMF solution for W^{NMF} (which can be calculated fairly quickly, using the NMF algorithm mentioned above). For step 2, we use a variant of the EM procedure we call EMcW, to estimate (A, B) while keeping W^{NMF} constant. This process requires the calculation of the ESS of $S^1, S^{\log}$ for the E-step, and the maximization of (A, B) for the M-step. We note that given W^{NMF}, the parameters (A, B) and the ESS of $S^1, S^{\log}$ are independent between features. Thus we can divide the features into batches and parallelize the EMcW procedure entirely.

Next, we use the resulting (A, B) parameters to determine which features are component-specific: We take $\left(\tilde{A}, \tilde{B}\right)$ to be the resulting (A, B) for the $\tilde{M} = 100 \cdot K$ features with the highest difference between the mean signal of different components (normalized by mean signal of the features). We then use these features in step 3 to adjust the starting point W^{NMF}, by running the EM algorithm to estimate the mixing weights W while keeping $\left(\tilde{A}, \tilde{B}\right)$ constant (EMcAB variant). This requires the calculation of the ESS of G, T in the E-step, and the maximization of W in the M-step, and results in $\hat{W}$ that is adjusted for component variation. Lastly, in step 4 we readjust the component parameters $\left(\hat{A}, \hat{B}\right)$ using the EMcW variant with the constant $\hat{W}$.

In our case we stop here and return the resulting $\hat{W}$ and $\left(\hat{A}, \hat{B}\right)$ from the last step. However, this alternation can clearly be repeated several times (steps 3 and 4). Since the EMcW variant is parallelized by batches and the EMcAB only uses $\tilde{M} = 100 \cdot K$ features, each iteration is much more efficient in both time and memory than running the regular EM procedure over a large number of features.

Normalization

A known limitation of the NMF model is that it is not identifiable: Say W, H are an NMF solution for dataset V. Then, for every invertible matrix J s.t.

$$W^{\mathrm{new}} \stackrel{\mathrm{def}}{=} W \cdot J \geq 0 \qquad H^{\mathrm{new}} \stackrel{\mathrm{def}}{=} J^{-1} \cdot H \geq 0 \tag{46}$$

we have

$$W^{\mathrm{new}} \cdot H^{\mathrm{new}} = (W \cdot J) \cdot \left(J^{-1} \cdot H\right) = W \cdot H \tag{47}$$

As the Poisson log-likelihood NMF depends only on the reconstruction $R = WH$ (Eq. 2), the cost of the two solutions will be identical, and therefore the KL-NMF problem is not identifiable.

This is important in a few ways: First, when measuring the quality of a solution, we want to compare the solution directly to ground truth parameters (if exist). We also compare two different solutions from different models. More crucially, we want,

for example, to be able to infer component patterns and use them to characterize states in the system (e.g. cell-types). If the components are not unique in a way that changes the real-life interpretation of them, it significantly harms the interpretability of the model.

We start by considering permutation and non-negative normalization matrices (diagonal matrices with non-negative elements). Since such matrices are invertible and their inverse is also non-negative [Plemmons and Cline, 1972], multiplying an NMF solution by them (as detailed in Eq. 47) will result in an equivalent solution. However, we can easily normalize NMF solutions to alleviate ambiguity of normalization and permutation and get *essentially unique* solutions that are comparable between models and runs: We use a reference solution to determine the order of the components (for synthetic data - according to the ground truth, for real data - some predefined order), and match the components order of the new solution using linear sum assignment. We then reorder the components in the solutions by multiplying H and W with a corresponding permutation matrix. For normalization, we normalize each component by a factor d_k by multiplying H and W with a corresponding normalization matrix. This results in an equivalent solution.

For VarNMF solutions we use the same process, except for the normalization of the components that require a different treatment: We want to scale the distributions of $\mathcal{H}$ by some factors $d_1, ..., d_K$. Following Lemma 5, we can simply scale the parameters B_k by the inverse d_k^{-1}, or equivalently multiply B by the inverse of the corresponding normalization matrix. This will result in an equivalent solution if we normalize W accordingly.

The choice of normalization depends on the data. For synthetic data, we normalize each component to have the same median value (over the features) as the matching ground truth component. For real data, we use a healthy reference — a vector that represents the typical signal of healthy samples (obtained from [Sadeh et al., 2021]). We choose a group of housekeeping genes, that are known to have high signal and low variation between cell-types and tissues (Sadeh et al.). We then normalize each component to have the same median value in these housekeeping genes as the healthy reference. This normalization procedure is meant to anchor all components' housekeeping genes (that have similar behavior in different cell-types) to a similar location.

Other matrices for which Eq. 46 holds can be used to scale only NMF and not VarNMF solutions, as it will cause different components to become correlated, thus the scaled solution will be distinct from the original solution (in which all features are independent). These matrices can sometimes exist, and the conditions for essential uniqueness of the NMF solutions are complicated [Donoho and Stodden, 2003]. Fortunately, in practice, following some basic sparseness properties, it is likely that NMF will have an essentially unique solution [Huang et al., 2013]. In our case, these conditions usually hold, and we will assume the NMF solution (normalized for permutations and normalization matrices) is unique.

Generalization

The train log-likelihood measure evaluates the ability of an algorithm to fit a dataset, and is a popular measure in the world of probabilistic modeling and unsupervised learning (which includes NMF research). However, in our case and many other situations, the overall goal is to extract an accurate distribution of features (e.g. genes). While the mixing weights are specific to each sample, the learned distributions are general and should fit all samples, including unseen ones. Therefore, we want to measure the generalization abilities of our model on a new dataset sampled using the same distribution.

To test the generalization abilities of VarNMF, we use the learned distributions to learn a new W^{test} for a test dataset V^{test} by applying EMcAB (Appendix 9.5). Similarly, for NMF, we use the learned constant components H to learn W^{test} by applying the NMF Multiplicative update rule [Lee and Seung, 2000] on V^{test} and keeping H constant. In both cases we report the test log-likelihood to be the log-likelihood for the test data with the train distributions or components and the test mixing weights.

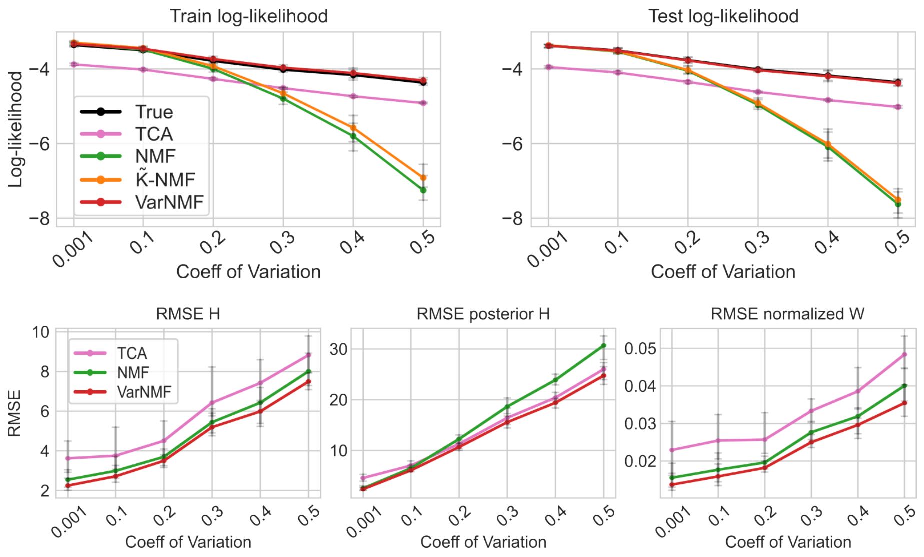

Fig. 10. Comparison to TCA: Decomposing synthetic data with increasing coefficient of variation. A) Train and test log-likelihood of the ground truth parameters and four models - TCA, VarNMF, NMF, $\tilde{K}$-NMF (NMF with higher degrees of freedom than VarNMF), versus the coefficient of variation of the dataset. The log-likelihood values are normalized to nats/observation. B-D) RMSE of the estimated parameters versus the ground truth: B) RMSE of the train and test weights estimated versus the ground truth weights. The weights are first normalized to 1 for every sample since the TCA weights are estimated on normalized data. C) RMSE of the mean of the distributions estimated by VarNMF and TCA and the constant components estimated by NMF versus the mean of the ground truth distributions. D) RMSE of the per-sample posterior profiles estimated by VarNMF and TCA and the constant components estimated by NMF versus the ground truth $\mathcal{H}[i]$. The shown values are the mean over T = 10 runs of datasets with K = 4 components and coefficient of variation cv = 0.2.

3. Synthetic data

Comparison to other decomposition methods

We conducted additional experiments to compare the performance of VarNMF and a previously published method, Tensor Composition Analysis (TCA, Rahmani et al. [2019]). As described above, this is a tensor decomposition model that learns the source distributions from data and estimates a per-sample component matrix $\mathcal{H}[i]$ for each sample i using the posterior estimation, similar to VarNMF. However, the TCA model assumes Normal source distributions with a non-negative mean (for which the posteriors can still contribute negative signals), while we focus on the non-negative Gamma distribution. Although our choice complicates the optimization process, it is a better fit for biological signals, where we assume only non-negative contributions from each source. To illustrate this point, we use the synthetic datasets with non-negative source contributions and increasing source variation from Section 4.1, and compare the models performances.

We ran the NMF, $\tilde{K}$-NMF, VarNMF and TCA on synthetic datasets with $N = 100$ samples, $M = 100$ features and $K = 4$ components. Since TCA requires normalized data as input, we normalized the dataset so that each sample signal sums (over the features) to 10^4. It also requires an estimation to W, which it then refits. We provided as a starting point the W that resulted from running NMF on the dataset (similar to the process in VarNMF).

In terms of log-likelihood, VarNMF outperforms TCA across all levels of variation (Fig. 10A). While this is not necessarily surprising, since the datasets were sampled from the generative model defined by VarNMF, this demonstrates the sensitivity of models like TCA to datasets with non-negative source contributions. Additionally, this advantage is retained in RMSE between the estimated parameters and the ground truth (Fig. 10B-D), suggesting an actual advantage of VarNMF in decomposing non-negative sources from mixed samples.

Results for different K values

We examine the results of NMF and VarNMF on synthetic datasets with increasing source variation and increasing number of sources (Section 4.1 for more details). The models are given the correct number of source K. In Fig. 11A we observe a decline in NMF train and test log-likelihood results as the levels of variation in the dataset (controlled by the coefficient of variation of the ground truth source distributions) increase. This is true for all values of K (#Components in the dataset) but is most apparent for small values of K. We conclude that VarNMF better captures the datasets distribution in the presence of high component variation.

Next, we examine the learned parameters of each model against the ground truth. Starting with the learned weights of NMF and VarNMF against the ground truth (Fig. 11B), the two models have almost identical values, with performances decreasing with the level of variation. As for learned components against the ground truth, the VarNMF component means are closer to the ground truth means than NMF's estimates (Fig. 11C) but generally the trends look similar: The RMSE values increase with the levels of variation and with the number of components. This suggests that component variation between samples increases the complexity of the data and hinders both algorithms efforts to extract the ground truth weights and mean of the sources distributions. However,

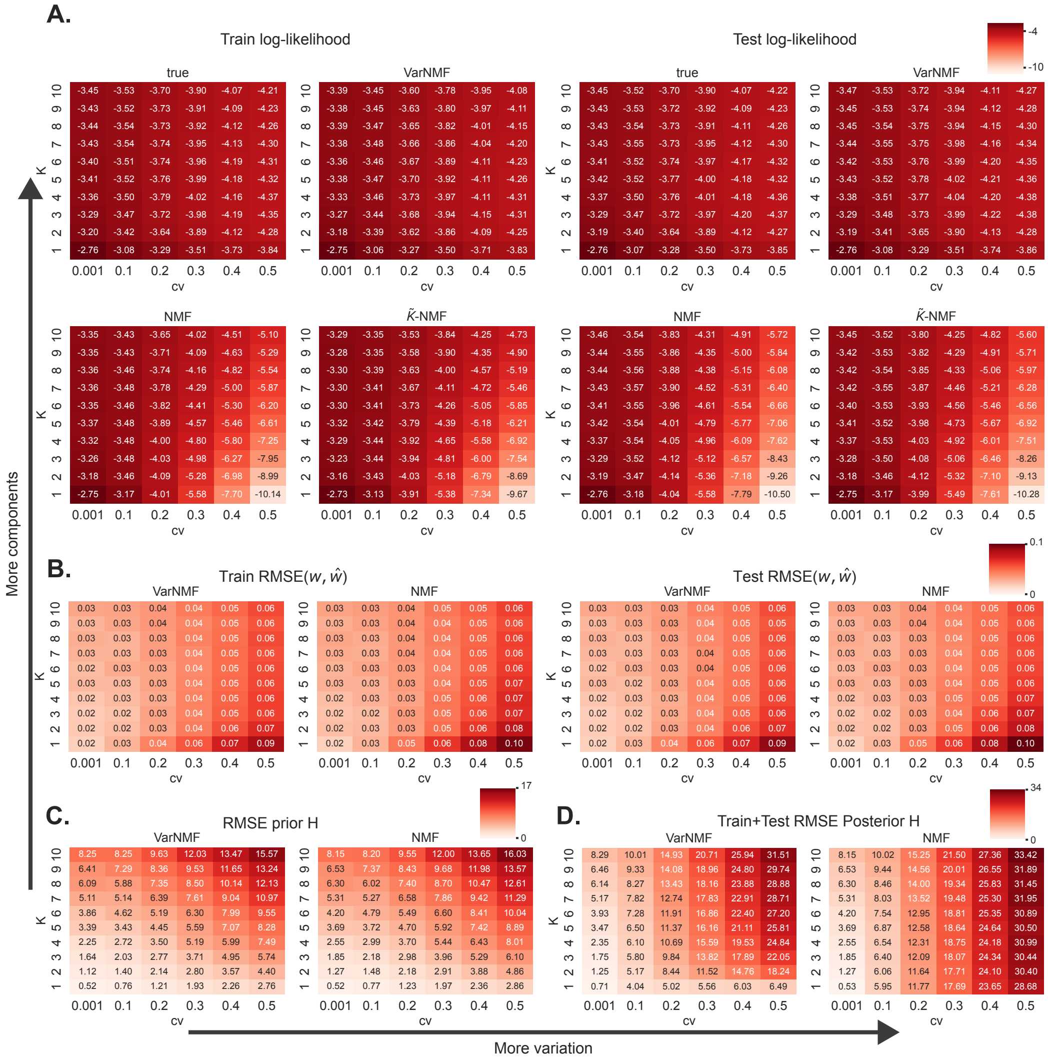

Fig. 11. Decomposing synthetic data with $K = 1, \ldots, 10$ components: A) Train and test log-likelihood of the ground truth parameters and three models - NMF, VarNMF and $\tilde{K}$-NMF (NMF with higher degrees of freedom than VarNMF), versus the coefficient of variation of the dataset and versus K. The log-likelihood values are normalized to nats/observation. B) Root mean square error (RMSE) of the train and test weights estimated by VarNMF and NMF versus the ground truth weights. C) RMSE of the mean of the distributions estimated by VarNMF and the constant components estimated by NMF versus the mean of the ground truth distributions. D) RMSE the per-sample posterior profiles estimated by VarNMF and the constant components estimated by NMF versus the ground truth $\mathcal{H}[i]$. The shown values are the mean over $T = 10$ runs.

comparing the ground truth per-sample contribution of source k, $\mathcal{H}[i]_k$, to the VarNMF posterior profiles versus NMF constant components (Fig. 11D), the VarNMF RMSE is similar to the NMF values for datasets with no variation (cv$= 0.001$), but outperform the NMF solution when this variation increases, and NMF perform poorly for every value of K.

Results with wrong K values

A worry in practice is that we would not have access to the correct number of components. To examine the sensitivity of the reconstruction to wrong values of K, we created synthetic datasets with trueK $= 4$ sources and increasing levels of source variation (coefficient of variation $= 0.001, 0.2, 0.5$, as in Section 4.1). We then decomposed these datasets using $K = 1, \ldots, 10$ components with NMF, VarNMF, and also NMF with additional components to compensate for difference in the number of free parameters, Fig 12. When K is small, All models suffer from big gaps in log-likelihood scores. These gaps decrease as K increases, and plateau at $K = 4 =$ trueK, as expected. However, in cases with higher source variation, both NMF models have a substantial gap from the ground truth, even for larger values of K. In contrast, VarNMF does not suffer from this gap and achieves the true log-likelihood using $K \geq 4$ components. This suggests that in settings with high source variation, VarNMF offers a better fit for the data than NMF, even when decomposing with the wrong number of components.

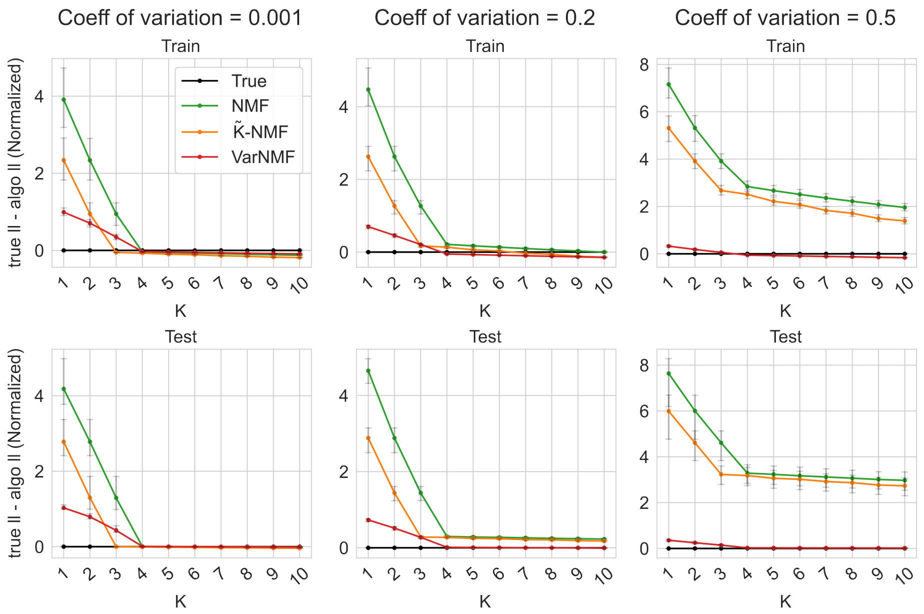

Fig. 12. Decomposing synthetic data with trueK $= 4$ sources using $K = 1, \ldots, 10$ components: Train (top) and test (bottom) log-likelihood of the ground truth parameters and three models - NMF, VarNMF and $\tilde{K}$-NMF (NMF with higher degrees of freedom than VarNMF, i.e. higher number of components), versus the number of components used in the decomposition, for three coefficient of variation values. The log-likelihood values are normalized to nats/observation. The shown values are the mean over $T = 10$ runs. Shown are the differences from true log-likelihood on the same data, to account for likelihood differences that arise from the data sampling process.

Robustness to sampling noise and numbers of samples and features

In Fig 13A,D we examine the effect of increasing sampling noise on the performances of NMF and VarNMF. This is done by increasing the limits of the uniform distribution from which we sample the per-sample scaling factors $\lambda[i]$ (Section 4.1 for more details). Specifically, applying VarNMF and NMF to datasets with scaling factors sampled from $\mathcal{U}\left(\left[\frac{l}{2}, l\right]\right)$ for $l = 2, 5, 10, 20$, results in an expected drop in performances. However, the NMF performances drop significantly more than those of VarNMF, suggesting that the advantage of VarNMF is robust to sampling noise.

Additionally, when increasing the number of samples N (Fig 13B,E) and features M (Fig 13C,F), we get similar or better estimations of the ground truth parameters, but also a consistent advantage of VarNMF over NMF. However, as mentioned above, the EM algorithm is computationally intensive. Therefore, for a very high number of features, we do not run it directly but rather run an Alternating EM version (Supplementary Section 9.5). This algorithm stops early and does not wait for convergence, but still achieves a good fit for the data distribution, and its estimation of the model parameters is better or comparable to NMF (Fig. 14). An alternative approach for a dataset with a large number of features is to apply a more aggressive feature selection procedure to lower the number of features to a manageable number. Using genes that are source-specific and stable across the population within the source, we can potentially learn a good estimation for the weights W, and later use this estimation to learn the source distributions over all genes.

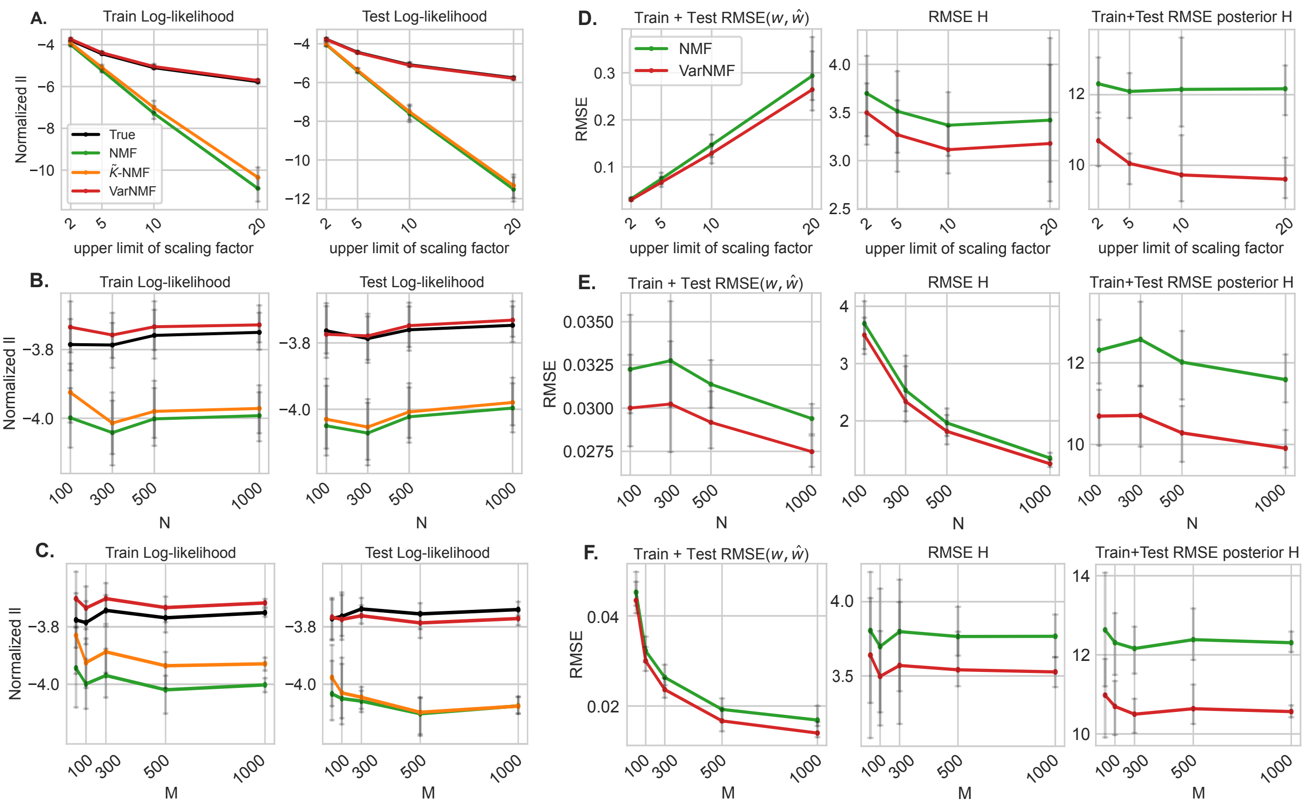

Fig. 13. Robustness to sampling noise (top), N (middle) and M (bottom): Decomposing synthetic data with increasing sampling noise, increasing number of samples and increasing number of features N: A-C) Train and test log-likelihood of the ground truth parameters and three models - NMF, VarNMF and $\tilde{K}$-NMF, A) versus the upper limit of the scaling factors distribution, B) versus the number of samples N, and C) versus the number of features M. The log-likelihood values are normalized to nats/observation. D-F) RMSE of the different parameters, D) versus the upper limit of the scaling factors distribution, E) versus the number of samples N, and F) versus the number of features M. Left - RMSE of the train and test weights estimated by VarNMF and NMF versus the ground truth weights. Middle - RMSE of the mean of the distributions estimated by VarNMF and the constant components estimated by NMF versus the mean of the ground truth distributions. Right - RMSE of the per-sample posterior profiles estimated by VarNMF and the constant components estimated by NMF versus the ground truth $\mathcal{H}[i]$. The shown values are the mean over $T = 10$ runs of datasets with $K = 4$ components and coefficient of variation cv $= 0.2$

	Train True LL - algo LL			Test True LL - algo LL			W RMSE		H RMSE		posterior H RMSE	
**CV**	**NMF**	**Ktilde-NMF**	**VarNMF**	**NMF**	**Ktilde-NMF**	**VarNMF**	**NMF**	**VarNMF**	**NMF**	**VarNMF**	**NMF**	**VarNMF**
**0.001**	**-0.02**	-0.05	**-0.02**	**0.03**	**0.03**	0.04	**0.02**	**0.02**	**3.48**	3.97	**3.48**	4.01
**0.2**	0.18	0.11	**-0.03**	0.29	0.30	**0.06**	**0.02**	0.03	**4.82**	4.87	12.81	**11.68**
**0.5**	2.48	2.19	**-0.04**	2.99	3.00	**0.08**	**0.02**	0.04	8.56	**8.20**	31.06	**26.17**

Fig. 14. Performances with alternating EM: Decomposing synthetic data with $M = 7000$ features, for three coefficient of variation values. Shown are the differences between the models' log-likelihoods from true log-likelihood on the same data (to account for likelihood differences arising from the data sampling process) and the RMSE of the different parameters versus the ground truth. The shown values are the mean over $T = 3$ runs of datasets with $K = 5$ components. Bold values indicate that the model estimation is the closest to the ground truth.

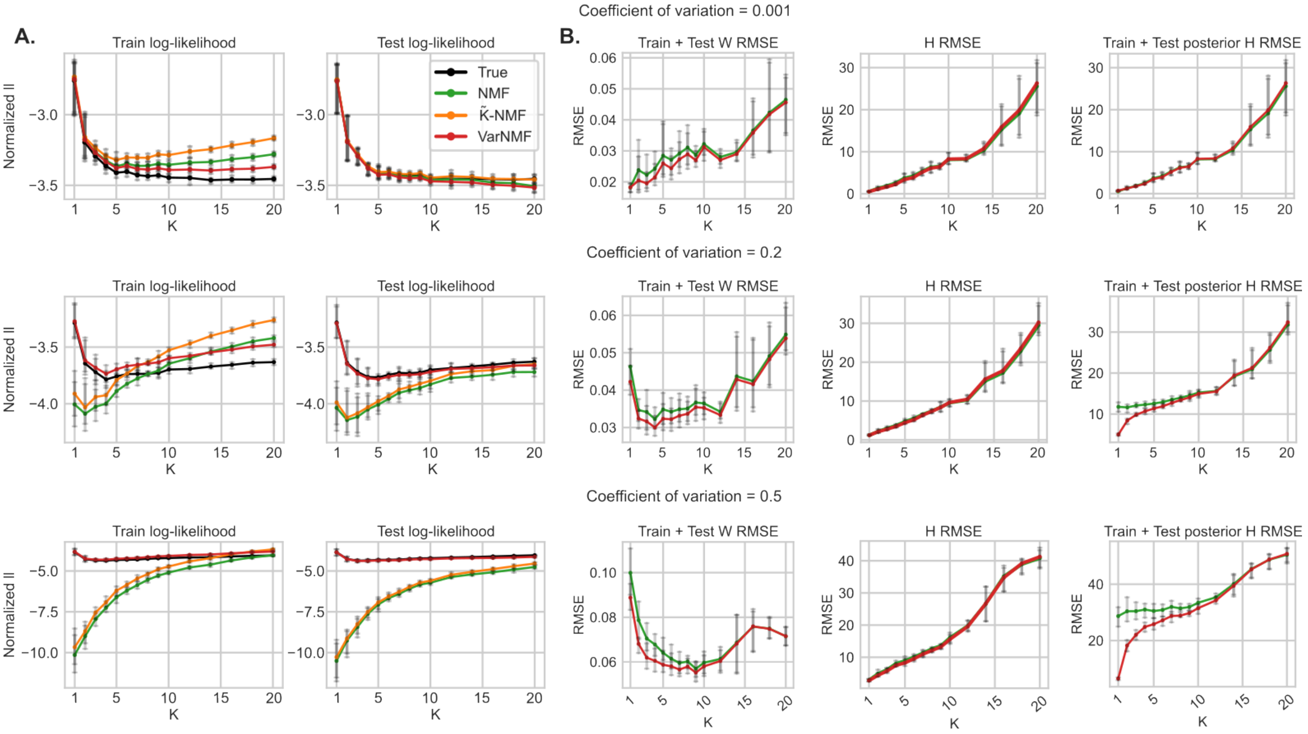

Fig. 15. Performances with a high number of components: Decomposing synthetic data with $1 \leq K \leq 20$ components, for three coefficient of variation values. A) Train and test log-likelihood of the ground truth parameters and three models - NMF, VarNMF and $\tilde{K}$-NMF versus K. B) RMSE of the different parameters versus K. The shown values are the mean over $T = 10$ runs.

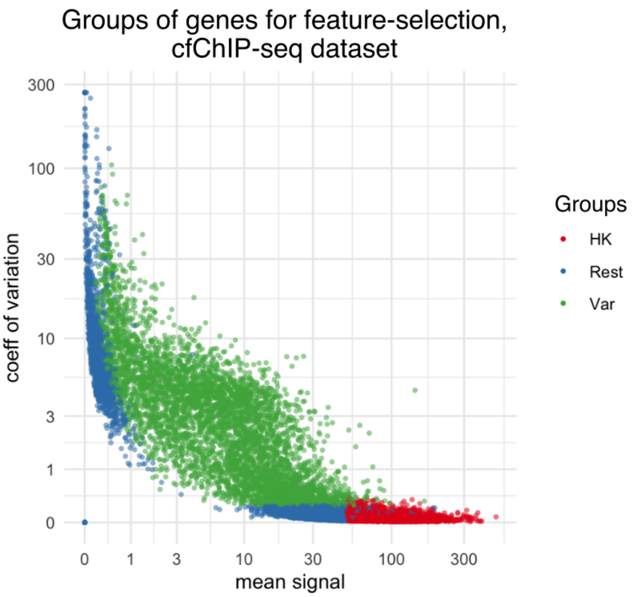

Fig. 16. Choosing genes for the cfChIP-seq dataset: Mean versus coefficient of variation for the normalized reads of the cfChIP-seq dataset [Sadeh et al., 2021]. Each point is a gene. The genes are divided into three groups - housekeeping-like genes (red), variable genes (green), and the rest (blue) - according to the thresholds described above.

4. cfChIP-seq data

We collected a dataset of cfChIP-seq samples from Sadeh et al. [2021] and Fialkoff et al. [2022]. This data includes plasma samples of 80 healthy subjects, 139 small-cell lung cancer (SCLC) patients, and 86 colorectal cancer (CRC) patients. This data has two representations.

- Normalized reads - gene values after normalization, According to Sadeh et al. [2021] the normalization ensures that the samples agree on a set of reference genes.
- Raw counts - gene values are the observed count in each sample.

We used the normalized reads representation for selecting genes (below) and for PCA analysis (Fig. 4). For the actual data processing we used raw counts, and let the model fit a scaling parameter per sample.

Choosing dataset features

To choose relevant features we first excluded genes that are positioned on chromosomes X or Y (sex-specific genes) and putative genes (ORFs without a name, pseudogenes and such).

For each gene we computed several statistics across the entire dataset — mean μ_g, variance σ_g^2, coefficient of variation $\eta_g = \sigma_g/\mu_g$, number of times they are above 0 n_g, and maximal value m_g. Based on these we defined three groups of genes (Fig. 16):

- Housekeeping-like genes [6029 genes] – Genes with high mean ($\mu_g > 50$), low variability ($\eta_g^2 < 0.5$), and without extreme outliers ($m_g < 3 \cdot \mu_g$).
- Variable genes [5119 genes] – genes that do not appear in the first group and have high variation ($\eta_g^2 > 0.25$), expression in more than 50 samples ($n_g > 50$), and some observations above a threshold ($m_g > 10$).
- Remaining non-excluded genes [5764 genes]

We reasoned that the housekeeping-like genes provide stability and anchor the estimation of values. The variable genes provide a chance to identify interesting phenomena. Thus we randomly selected 5000 variable genes, and added 1000 randomly selected genes from each of the two other groups.

Testing gene-lists versus curated databases

To choose genes that differ in one component k from the rest, we first discard genes that have a value lower than 10 in the specific component (or in the mean of the component, in the case of VarNMF). We then calculate the median value across all components for each gene, and sort the genes in descending order based on the fold-change increase observed in component k relative to the calculated median. Genes with less than a 2-fold increase are excluded.

Next, we test whether these genes are significantly over-represented in curated genes-lists associated with a specific tissue or cell-type (using the Enrichr tool, Chen et al. [2013] for details). The top results for each component of the NMF and VarNMF solutions with $K = 4$ components are presented in Tables 1, 2. For the "healthy" components we used reference data from human

Table 1. Enrichr results for NMF solution, $K = 4$

Component	Database	Term	Overlap	Adj. p-value
1	BioPlanet 2019	Hemostasis pathway	12/468	3.52e-09
37 genes	BioPlanet 2019	Platelet activation signaling and aggregation	8/205	2.19e-07
2	ARCHS4 Tissues	PERIPHERAL BLOOD	165/2316	8.93e-75
297 genes	ARCHS4 Tissues	NEUTROPHIL	140/2316	9.49e-52
	ARCHS4 Tissues	MACROPHAGE	137/2316	1.88e-48
3	Cancer Cell Line Enc.	CORL279 LUNG	116/465	1.10e-84
464 genes	Cancer Cell Line Enc.	CORL24 LUNG	117/569	1.98e-75
	Cancer Cell Line Enc.	NCIH446 LUNG	94/327	3.20e-74
4	Cancer Cell Line Enc.	SNU283 LARGE INTESTINE	58/189	5.99e-55
329 genes	Cancer Cell Line Enc.	CL40 LARGE INTESTINEE	55/210	5.63e-48
	Cancer Cell Line Enc.	SW1463 LARGE INTESTINE	49/177	6.22e-44

Table 2. Enrichr results for VarNMF solution, $K = 4$

Component	Database	Term	Overlap	Adj. p-value
1	Reactome 2022	Hemostasis	10/576	3.11e-07
28 genes	BioPlanet 2019	Hemostasis pathway	11/468	2.12e-09
	BioPlanet 2019	Platelet Activation, Signaling and Aggregation	8/205	1.91e-08
2	ARCHS4 Tissues	PERIPHERAL BLOOD	86/2316	1.24e-52
118 genes	ARCHS4 Tissues	NEUTROPHIL	76/2316	1.45e-40
	ARCHS4 Tissues	GRANULOCYTE	71/2316	3.56e-35
	ARCHS4 Tissues	MACROPHAGE	66/2316	4.05e-30
3	Cancer Cell Line Enc.	CORL279 LUNG	155/465	8.09e-108
700 genes	Cancer Cell Line Enc.	NCIH446 LUNG	122/327	1.50e-90
	Cancer Cell Line Enc.	CORL24 LUNG	150/569	6.80e-88
4	Cancer Cell Line Enc.	JHH5 LIVER	89/245	5.58e-88
383 genes	Cancer Cell Line Enc.	C3A LIVER	95/387	3.12e-76
	Cancer Cell Line Enc.	SNU283 LARGE INTESTINE	53/189	9.57e-45
	Cancer Cell Line Enc.	SW1463 LARGE INTESTINE	51/177	1.03e-43

tissues and cell-types (*Reactome 2022*, *BioPlanet 2019* and *ARCHS4 Tissues*). For the disease-associated components, we made use of the *Cancer Cell-Line Encyclopedia* (CCLE) which contains data from cells of various cancers.

Results for the first three components are similar between the two models. The first two components' genes are strongly enriched for Platelets and Neutrophils, which are the two main sources of cell-free DNA in healthy samples [Sadeh et al., 2021]. The second component is also enriched for Macrophages that differentiate from Monocytes which are also found in high concentrations in cell-free DNA from healthy samples. The third component (with non-zero weights mostly in SCLC patients) is enriched specifically for SCLC-derived cell-lines in both the NMF and VarNMF solutions. This indicates that this component indeed represents the tumor-derived cell-free DNA in the SCLC patients plasma. The fourth component (with non-zero weights mostly in CRC patients) displays different associations between the two models: The NMF solution is enriched exclusively for colon-derived cell-lines, aligning with the CRC patients diagnosis. On the other hand, the VarNMF solution is enriched for both liver and colon derived cell-lines. The former enrichment may reflect liver metastasis which exists in many of the CRC patients in this cohort.

Results of NMF and VarNMF for a range of Ks

We examine the decomposition of the NMF and VarNMF models and algorithm on cfChiP-seq dataset with increasing number of components $K = 2, \ldots, 6$ (Section 4.2 for more details). In Fig. 18 we look at the resulting weights. We observe similar results in the train and test datasets as well as for both NMF and VarNMF. However, in the NMF solution we observe a single healthy component (that is high in healthy samples but also appears in a range of weights in the cancer samples) whereas VarNMF displays two. Additionally, starting from $K = 3$, there are at least two components that are cancer-specific (that have non-zero weights

mostly in one cancer type). The one SCLC-associated component of $K = 4$ splits into two SCLC-associated components starting from $K = 5$, and the CRC-associated component remains unique in all solutions.

Lastly, we observe a shared-cancer component in the $K = 4, 5$ solutions. In VarNMF it is also shared with healthy samples and is associated with genes of blood-related cell-types, which is expected (Supplementary Section 11.2). In NMF, however, this component is not shared with healthy samples, but is still associated with these cell-types. Starting from $K = 6$, the two models result in two healthy components, and one shared cancer component. This suggests that both models extract a component that represents shared features between the cancers.

We conclude that both VarNMF and NMF result in similar weights, with one or two healthy components that reflect the healthy cell-free material in the plasma, one or two cancer-specific components for each type of cancer, that represent the disease contribution to the plasma, and possibly one component that is shared between the cancers.

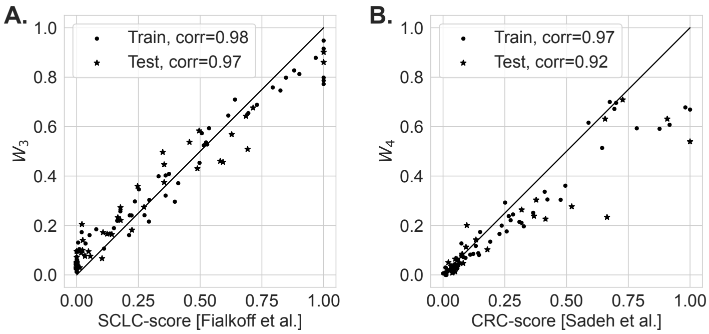

Fig. 17. Correlations of the weights estimated by VarNMF for components #3 (A) and #4 (B) versus supervised estimations of disease load [Fialkoff et al., 2022, Sadeh et al., 2021]

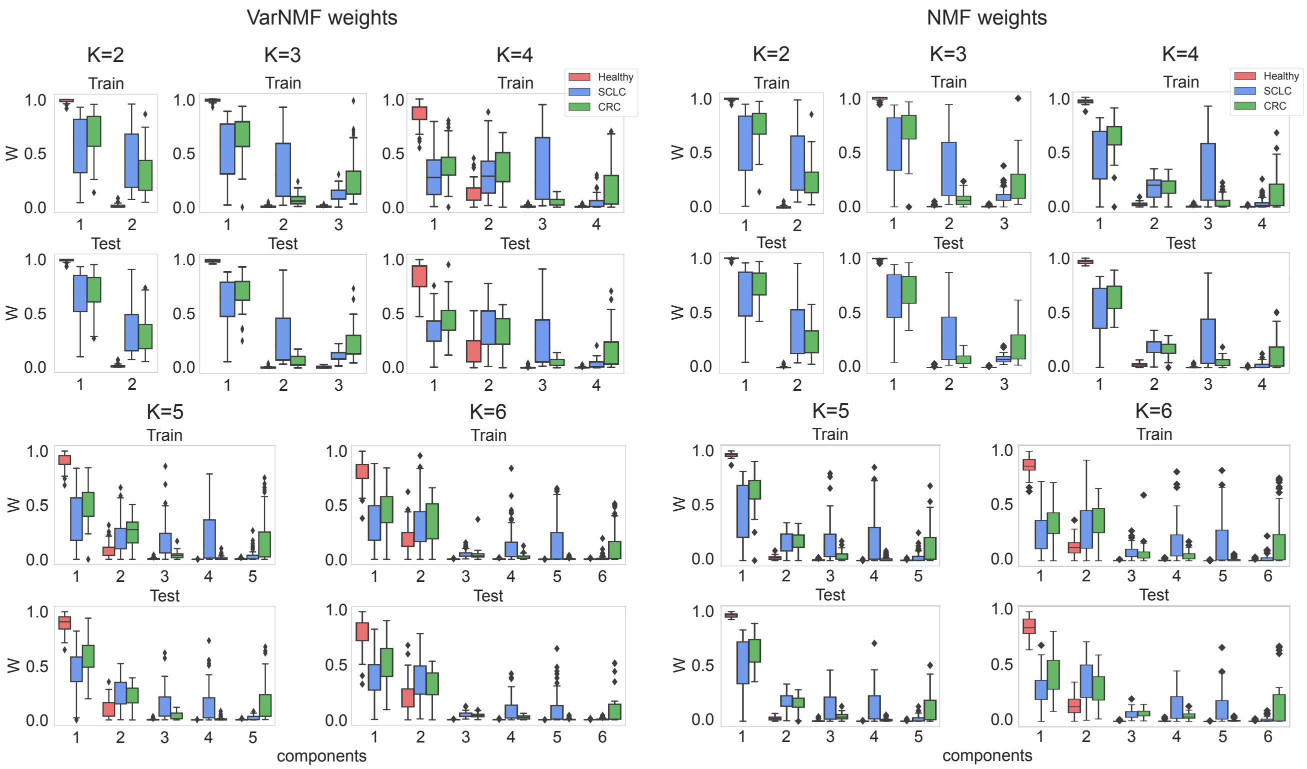

Fig. 18. Decomposing cell-free ChIP-seq data with VarNMF (left) and NMF (right): The estimated train and test weights for each component in the $K = 2, \ldots, 6$ solutions, aggregated by sample sub-cohort (healthy, SCLC and CRC). Weights are normalized so that each sample has a total weight of 1.

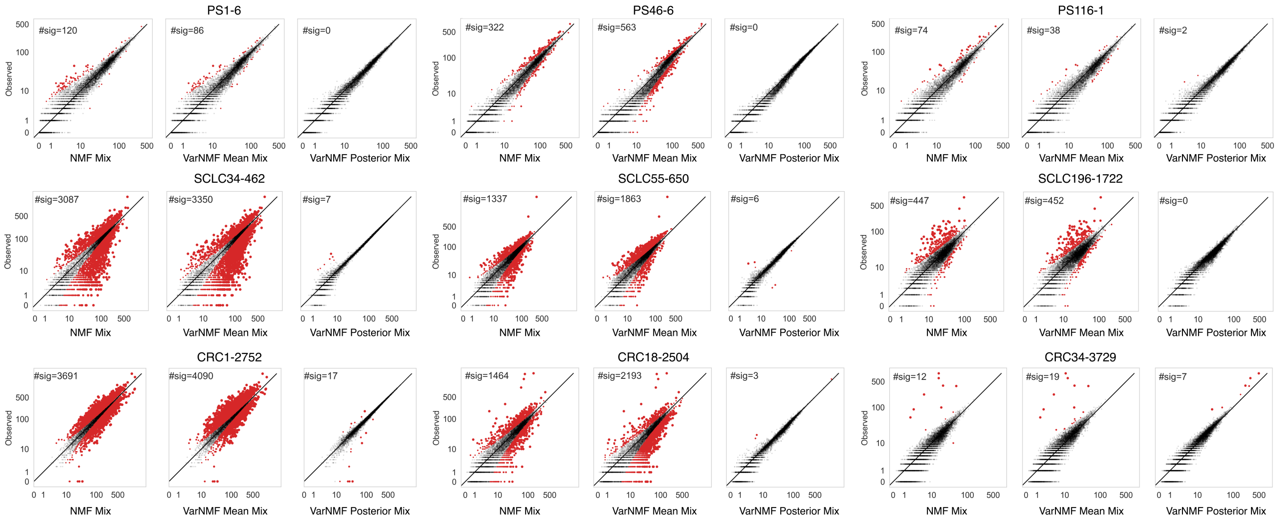

Fig. 19. Reconstructing H from data with the $K = 4$ solution: Reconstruction quality of representative samples by NMF (left), VarNMF mean components (middle), and VarNMF posteriors (right). Each point is a gene, the x-axis shows the reconstructed value R and the y-axis shows the observed value V. Red points are ones that are significantly different, accounting for Poisson sampling noise (q-values corrected for false discovery rate - < 0.05).

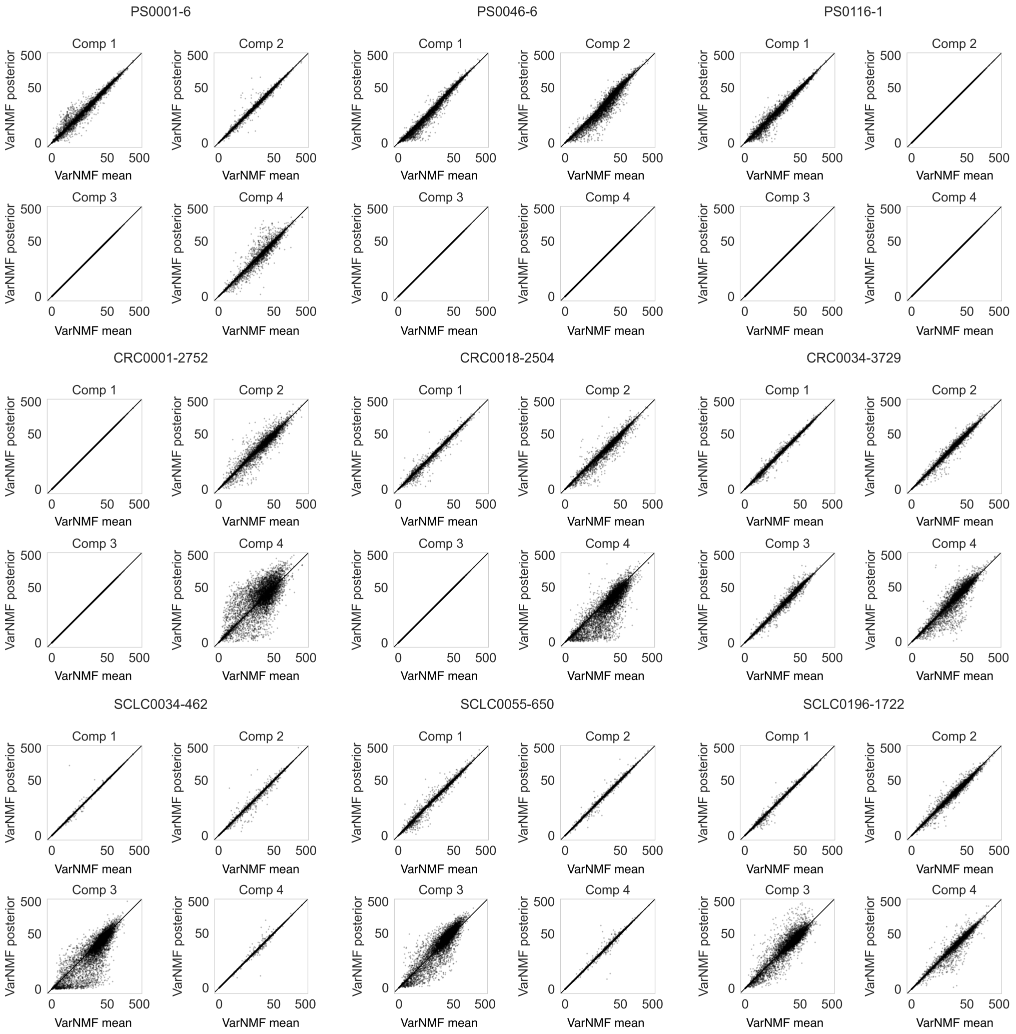

Fig. 20. Reconstructing H from data with the $K = 4$ solution: For the same examples as in Supplementary Fig. 19, the component mean vs. posterior profiles per component.

5. Pseudo bulk RNA-seq data

Datasets

As an additional illustration of VarNMF performances, we apply it to pseudo-bulk RNA-seq data. This data is designed to simulate actual bulk RNA data, while also enabling comparison to a ground truth. To create pseudo-bulk samples, we used a publicly available dataset of RNA-seq samples from immune cell types, isolated from blood of healthy individuals [Calderon et al., 2019]. This dataset contains 166 normalized RNA-seq samples with or without immune induction, from six lineages - B, CD4, CD8, NK, Myeloid, and Gamma-Delta (GD) cells. Each lineage contains several cell-types (e.g., naive CD4 T cells, memory CD4 T cells). Note that while all six lineages contain immune cells, some are more distinct than others. We defined six sources of signal using the original paper's labeling of lineages. We used only control samples (without immune induction). From each lineage, we discarded cell-types that have less than 4 samples. This results in an atlas of 68 RNA-seq samples from six lineages, with 4-31 samples from each lineage. We denote the RNA samples from the k-th lineage as RNA$[k]$.

We created two datasets of mixed pseudo-bulk RNA samples: one with contributions from four distinct lineages - B, CD4, NK, and Myeloid - and one with all six available lineages (we denote the true number of lineages in the dataset K_{true}). Creating the datasets was done similarly to the synthetic data experiments: For each pseudo-bulk sample i, we first constructed its sample-specific profile $\mathcal{H}[i]_k$ for the k-th lineage by sampling 3 RNA samples from RNA$[k]$ and summing their expression per gene. This sampling process is meant to capture the biological variation between individuals in a lineage RNA signal. Next, we mixed these profiles using a weight vector $W[i] \in \mathbb{R}^{K_{\text{true}}}$ drawn from a symmetric Dirichlet (1) distribution, and normalized the results to sum (over the genes) to 10^7, to achieve a constant "sequencing-depth" across the samples. We then sampled the observation $V[i]_j$ for each gene $j = 1, \ldots, 19015$ from a Poisson with the normalized mixture as its rate.

We used the RNA atlas to select features for these datasets. The original datasets contained 19015 genes, from which we chose 2300 genes in the case of the 4-lineages dataset and 2800 genes for the 6-lineages dataset. First, due to the high dynamic range of RNA data, there are genes in the atlas with very high expression. To avoid high computational costs during training, we filtered out genes with high maximum levels in the atlas (max > 3000) and filtered out the 5% highest genes in the dataset. Additionally, we filtered out genes that had low values across the entire atlas (max ≤ 10). This left us with 8691 genes. Finally, we divided the genes into three gene sets, that were chosen according to the lineages' mean and variance in the atlas (we describe the number of genes for each group in the $K_{\text{true}} = 6$ case, the quantities for $K_{\text{true}} = 4$ are similar):

- Housekeeping-like genes [1204 genes, from which we randomly chose 500] – Genes with high mean levels in the atlas (mean > 100) and low variability ($\text{CV}^2 < 1.5$).
- Differential genes [2218 genes, from which we randomly chose 2000] – genes that had high levels in one lineage compared to the median of the lineages (log fold change > 2).
- Remaining non-excluded genes that had high mean levels in the atlas (mean > 100) [2008 genes, from which we randomly chose 300]

Results

Decomposing both datasets using VarNMF, NMF, and NMF with a comparable number of parameters ($\tilde{K}$-NMF) to $K = 2, \ldots, 10$ components, we get a significant advantage for VarNMF in terms of train and test log-likelihood across all values of K (Fig. 21A). This illustrates again that VarNMF results in a better fit of mixed data, that generalizes to new samples. However, the log-likelihood increases with K and does not achieve a maximum point in the true number of sources K_{true} for any of the models. This could be a consequence of high variation in the lineages signals, which is better characterized by a greater number of components.

Nevertheless, we investigate the parameters of some of the solutions to examine the algorithm's ability to accurately capture sources signals. For the 4-lineages dataset, the $K = 4$ solution identifies the mean of all four lineages in both the NMF and VarNMF models, with better RMSE scores for VarNMF (Fig. 21B,C). VarNMF also learns more accurate posterior profiles for this dataset.

As for the 6-lineages dataset, the $K = 6$ solution of both algorithms does not identify some of the six contributing lineages (Fig. 22A,B). Instead, they identify three district lineages – B, MEYLOID and NK – and three components for the T-cell contribution (CD4, CD8 and GD lineages, broken down not by this classification, Fig. 22C). This is consistent with the large diversity in T cell states and the parallels between CD4+ and CD8+ cells.

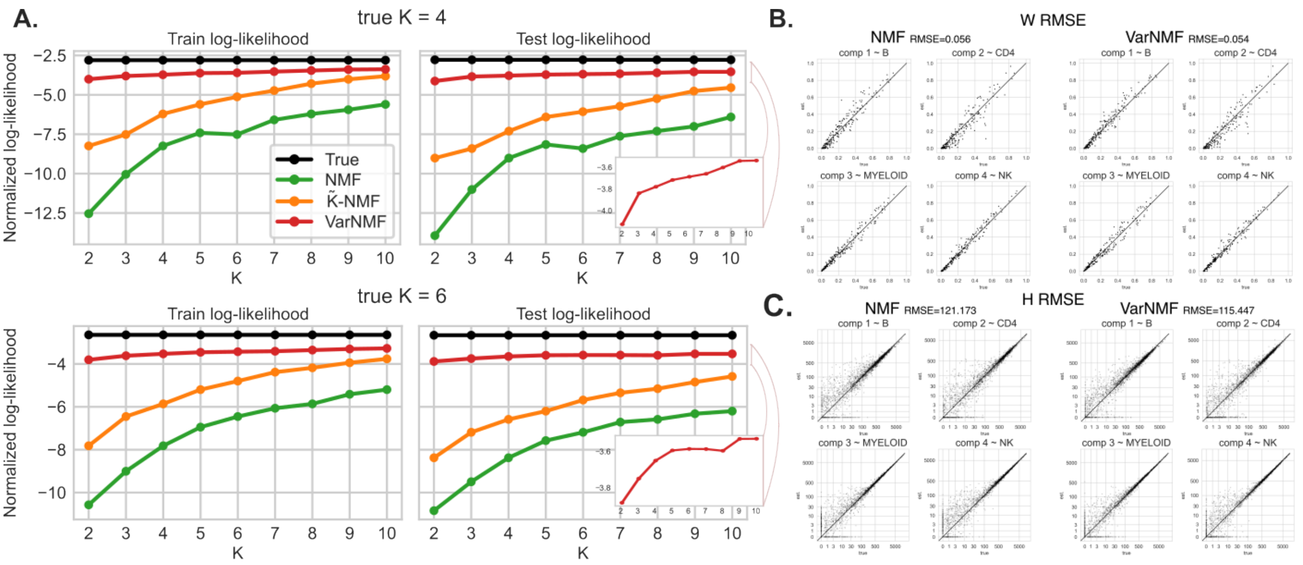

Fig. 21. Decomposition of pseudo-bulk RNA-seq: Decomposing the two RNA datasets with $2 \leq K \leq 10$ components. A) Train and test log-likelihood of the ground truth model (calculated using the true posterior profiles of each sample) and three models - NMF, VarNMF and $\tilde{K}$-NMF versus K. B,C) For the 4-lineages dataset - Scatter plots of the ground truth versus the NMF and VarNMF estimated W (B) and H (C). The values of W are normalized so that the weights of each sample sum to 1.

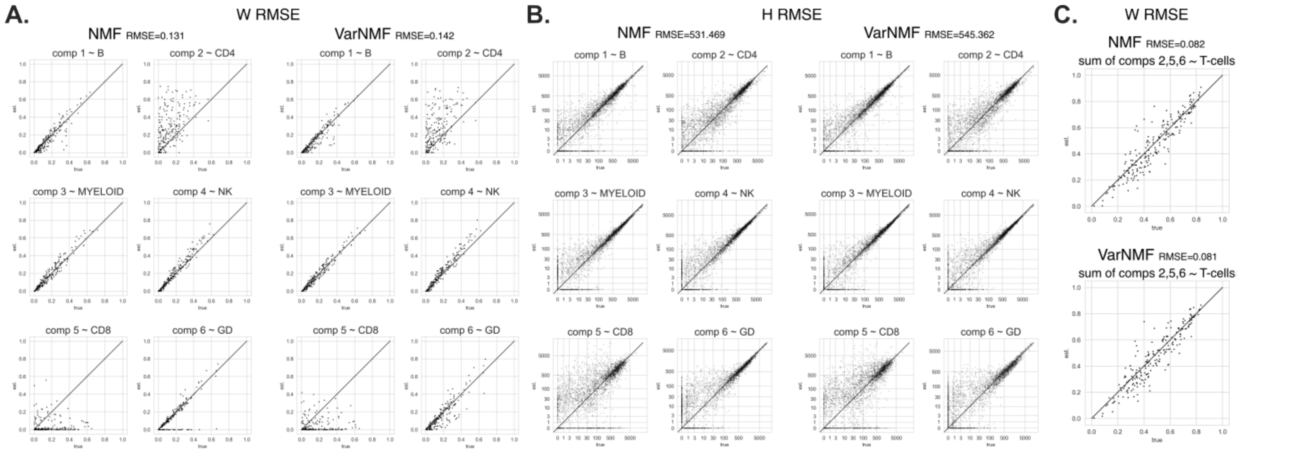

Fig. 22. Decomposition of pseudo-bulk RNA-seq with 6-linages: A,B) Scatter plots of the ground truth versus the NMF and VarNMF estimated W (A) and H (B). C) Scatter plots of the aggregated ground truth weights of all T-cell lineages (CD4, CD8 and GD) versus the aggregated estimated W of components 2,5,6. The values of W are normalized so that the weights of each sample sum to 1.
